# Supplementary material for: Epidemiology and transmission dynamics of multidrug-resistant organisms in nursing homes within the United States
Source: Nat Commun. 2025 Mar 13;16:2487. doi: 10.1038/s41467-025-57566-3 (PMC11906779; doi:10.1038/s41467-025-57566-3)
Supplement: Supplementary file 1 — Supplementary information [file 41467_2025_57566_MOESM1_ESM.pdf]

EPIDEMIOLOGY AND TRANSMISSION DYNAMICS OF MULTIDRUG-RESISTANT ORGANISMS IN NURSING HOMES WITHIN THE UNITED STATES: SUPPLEMENTARY INFORMATION

SUPPLEMENTARY TABLES

Supplementary Table 1. Significant characteristics of Veterans Affairs nursing home participants, by colonization status at baseline.

	**Overall (n = 188)[a]**	**Colonized at Baseline (n = 68)**	**Not Colonized at Baseline (n = 120)**	**P-value**
Recent Antibiotic Use	129 (68.6%)	57 (83.8%)	72 (60.0%)	7.2e-4
Wounds at Baseline	64 (34.0%)	30 (44.1%)	34 (28.3%)	0.028
Length of Preadmission Hospitalization, days				0.002
0–3 days	32 (17.0%)	5 (7.4%)	27 (22.5%)	
4–7 days	51 (27.1%)	16 (23.5%)	35 (29.2%)	
8–14 days	59 (31.4%)	21 (30.9%)	38 (31.7%)	
>14 days	46 (24.5%)	26 (38.2%)	20 (16.7%)	
Recent hospitalization length, days, median (IQR)	8.5 (5-14)	13 (7-18)	7 (4-12)	1.3e-4
	**Facility A (n=94)**	**Colonized at Baseline (n=33)**	**Not Colonized at Baseline (n=61)**	**P-value**
Recent Antibiotic Use	73 (77.7%)	31 (93.9%)	42 (68.9%)	0.005
Recent hospitalization length, days, median (IQR)	10.0 (4.0-15.0)	13.0 (7.0-18.0)	8.0 (2.0-13.0)	0.030
Katz (ADL) Score [b], median (IQR)	3.6 (2.3)	4.5 (2.0)	3.1 (2.3)	0.005
Functional dependence [c] at baseline				
Dependence in Transferring	70 (74.5%)	29 (87.9%)	41 (67.2%)	0.046
Dependence in Bathing	67 (71.3%)	29 (87.9%)	38 (62.3%)	0.009
Dependence in Toileting	64 (68.1%)	27 (81.8%)	37 (60.7%)	0.036
Dependence in Dressing	60 (63.8%)	26 (78.8%)	34 (55.7%)	0.026
Dependence in Continence	45 (47.9%)	22 (66.7%)	23 (37.7%)	0.007
	**Facility B (n=55)**	**Colonized at Baseline (n=24)**	**Not Colonized at Baseline (n=31)**	**P-value**
Recent Antibiotic Use	32 (58.2%)	19 (79.2%)	13 (41.9%)	0.006
Recent hospitalization length, days, median (IQR)	10.0 (6.0-14.0)	13.0 (7.5-19.5)	8.0 (5.0-12.0)	0.020
	**Facility C (n=39)**	**Colonized at Baseline (n=11)**	**Not Colonized at Baseline (n=28)**	**P-value**
Length of Preadmission Hospitalization				0.028
0–3 days	10 (25.6%)	2 (18.2%)	8 (28.6%)	
4–7 days	14 (35.9%)	3 (27.3%)	11 (39.3%)	
8–14 days	6 (15.4%)	0 (0.0%)	6 (21.4%)	
>14 days	9 (23.1%)	6 (54.5%)	3 (10.7%)	

Data are number of participants (%), unless otherwise specified. Two-sided p-values are based on ANOVA for continuous variables and Pearson's chi-square or Fisher's exact test for categorical variables. Two-sided p-value for "Recent hospitalization length," is based on Kruskal-Wallis test.

Abbreviations: ADL, activities of daily living; IQR, interquartile range; PICC, peripherally inserted central catheter.

[a]Due to data missing on admission. Total n=188 participants due to missing data on nine participants: race (n=7 missing) and hospital length of stay (n=2 missing).
[b]Overall Katz score ranges from 0-6, with 0 being independent and 6 being dependent in all six ADLs assessed.
[c]Dependence in function are defined as (1) transferring: needs help in moving from bed to chair or requires a complete transfer; (2) bathing: needs help with bathing more than one part of the body, getting in or out of the tub or shower, or requires total bathing assistance; (3) toileting: needs help transferring to the toilet, cleaning self or uses bedpan or commode;(4) dressing: needs help dressing self or needs to be completely dressed; (5) continence: is partially or totally incontinent of bowel or bladder.

Supplementary Table 2. Significant characteristics of Veterans Affairs nursing home participants, by acquisition of new MDROs.

	**Overall (n = 173)[a]**	**New MDRO Acquired (n = 71)**	**New MDRO Not Acquired (n = 102)**	**P-value**
Uses PICC Line	35 (20.2%)	19 (26.8%)	16 (15.7%)	0.074
Length of stay in study, days, median (IQR)	28 (15-42)	34 (21-54)	25.5 (12-36)	0.002
	**Facility A (n=88)**	**New MDRO Acquired (n=33)**	**New MDRO Not Acquired (n=55)**	**P-value**
Uses PICC Line	20 (22.7%)	12 (36.4%)	8 (14.5%)	0.018
Length of stay in study, days, median (IQR)	29.0 (15.5-40.0)	33.0 (22.0-45.0)	27.0 (13.0-36.0)	0.020
	**Facility B (n=51)**	**New MDRO Acquired (n=23)**	**New MDRO Not Acquired (n=28)**	**P-value**
Length of stay in study, days, median (IQR)	35.0 (20.0-60.0)	37.0 (22.0-77.0)	31.5 (17.5-39.0)	0.054
	**Facility C (n=34)**	**New MDRO Acquired (n=15)**	**New MDRO Not Acquired (n=19)**	**P-value**
No significant variables				

Data are number of participants (%), unless otherwise specified. Two-sided p-values are based on ANOVA for continuous variables and Pearson's chi-square or Fisher's exact test for categorical variables. Two-sided p-value for “Recent hospitalization length,” is based on Kruskal-Wallis test.

Abbreviations: IQR, interquartile range; PICC, peripherally inserted central catheter.

[a]Due to data missing on admission. Total n=173 participants due to missing data on 8 participants: race (n=7 missing) and hospital length of stay (n=1 missing)-- and excluding 16 participants (12 discharged after enrollment visit and 4 colonized with all MDROs at baseline).

Supplementary Table 3. Categorization of participants for sequencing selection, based on MDRO colonization, room contamination, new acquisition, and transmission.

						VRE		MRSA	
Category	**Participant colonized on ≥1 in-room visit**	**Participant room contaminated on ≥1 in-room visit**	**Participant with contamination at any interactive visit**	**Participant with transmission during an interactive visit**	**Participant with new acquisition during follow-up**	**No. Participants**	**No. Isolates**	**No. Participants**	**No. Isolates**
1	No	No	No	No	No	65	0	130	0
2	Yes	No	No	No	No	9	10	3	3
3	Yes	Yes	No	No	--	46	398	12	170
3b	Yes	Yes	No	No	Yes	21	105	6	65
4	Yes	Yes	Yes	--	--	34	622	12	227
4b	Yes	Yes	Yes	Yes	--	22	450	8	144
4c	Yes	Yes	Yes	Yes	Yes	6	81	2	14
5	No	Yes	No	No	No	32	54	23	29
6	No	No	Yes	--	No	4	7	11	12
7	Yes	No	Yes	--	--	1	2	3	8
8	No	Yes	Yes	--	No	6	23	3	9
Total						197	1116	197	458

Abbreviations: MDRO, multidrug-resistant organism; VRE, vancomycin-resistant enterococci; MRSA, methicillin-resistant *Staphylococcus aureus*.

Supplementary Table 4. Veterans Affairs nursing home characteristics and infection control policies.

	VA Facility		
**VA Characteristics** (as of Dec 2022)	**A**	**B**	**C**
No. Inpatient Beds	106	646	432
Skilled Nursing Beds	46	174	115
**Infection Control (IC) Program & Infrastructure**			
FTE dedicated to IC activities	3.5	5	3
FTE dedicated to antimicrobial stewardship	1.5	2.5	1.3
Does your facility have a specified person (e.g., staff, consultant) who is responsible for coordinating the IC program?	Yes	Yes	Yes
What level of professional training does he or she have?	Registered Nurse (RN)	Physician	Physician
How many years of IC experience does this person have at this facility?	≥1 - < 3 years	≥10 years	≥10 years
How many years of IC experience overall does this person have?	≥1 - < 3 years	≥10 years	≥10 years
Has this person received training in IC?	No	Yes	Yes
Does your facility have a process for reviewing infection surveillance data and infection prevention activities (e.g., presentation at QA committee)?	Yes	Yes	Yes
Does your facility have written infection control policies and procedures available and based on evidence-based guidelines, regulations, or standards?	Yes	Yes	Yes
Does your facility have a process for making policy changes at a local level?	Yes	Yes	Yes
Does your facility have an environmental cleaning policy implemented facility wide?	Yes	Yes	Yes
Does your facility have an environmental cleaning policy specific within each department?	Yes	Yes	No
Who provides IC-related training to the staff at your facility?	Infection control nurse and MDRO coordinator.	Infection control nurse, MDRO coordinator, and antimicrobial stewardship lead	Infection control nurse, MDRO coordinator, antimicrobial stewardship lead, and nursing education department
**MDRO Surveillance and Disease Reporting**			
Does your facility have written intake procedures to identify MDRO-colonized or infected persons at the time of NH admission?	Yes	Yes	Yes
Does your facility have a system in place in your NH for notification of IC coordinator when an antibiotic-resistant organism or C. difficile are reported by the clinical laboratory?	No, we don't have an active process but the MDRO coordinator can go in and manually look.	Yes, use both Theradock and through Vista and CPRS	Yes, the lab contacts the NH so patients can be isolated. IC uses data extraction tools, electronic reporting via corporate data warehouse to create daily reports.
Does your facility have a written surveillance plan outlining the activities for monitoring/tracking infections occurring in residents of the NH?	Yes	Yes	Yes
Does your facility have a system to follow-up on clinical information (e.g., laboratory, procedure results and	Yes	Yes	Yes

diagnoses), when residents are transferred to acute care hospitals for management of suspected infections, including sepsis?			
We track NH's current MRSA infection rates	Yes	Yes	Yes
We track NH's current Hand hygiene rates	Yes	Yes	Yes
We track NH's current gown and glove use rates	No	Yes	No
**Hand Hygiene (HH)**			
Do your NH's hand hygiene policies promote preferential use of alcohol-based hand rub (ABHR) over soap and water in most clinical situations, except in certain cases (e.g., C. difficile infections, norovirus, etc)?	Yes	Yes	Yes
Do all NH personnel receive training and competency validation on HH at the time of employment?	Yes	Yes	Yes
Does your NH routinely audit (monitor and document) adherence to HH?	Yes, patient safety performs auditing	Yes, HH monitors that report back to personnel and management	Yes, unit staff do secret evaluation and that data is reported to the Joint Commission Tracers with AMP® program
Does your NH provide feedback to personnel regarding their HH performance?	Yes, data presented to IC committee and other committees which is communicated to staff to a degree	Yes, it is reported to management and shared at staff meetings. Repeat offenders are given education as needed.	Yes, some corrections provided in real-time
Are supplies necessary for adherence to HH (e.g., soap, water, paper towels, ABHR) readily accessible in resident care areas (i.e., nursing units, resident rooms, therapy rooms)?	Yes	Yes	Yes
**Personal Protective Equipment (PPE)**			
Does your facility perform job-specific training and competency validation for personnel on proper use of PPE at the time of employment?	Yes, there is verbal training for new employees (especially nurses, CNAs, etc.). Specific steps are now taken for COVID-19, which includes central education for a trainee. There is new employee training for 2 days for all employees, but new physicians often mis it.	Yes, during general orientation done by the MDRO coordinator.	Yes, through nursing education and orientation
Does your facility perform job-specific training and competency validation for personnel on proper use of PPE annually?	Yes, there are patient safety fairs that are more virtual/remote due to COVID-19.	Yes, there is an annual TMS module and facility-wide PPE training/review	Yes
Does your facility perform routine audits (monitors and documents) of adherence to PPE use (e.g., adherence with indicated, donning/doffing)?	Yes, although only on the inpatient side. There is not one for NHs.	Yes, our program allows us to specify job area, discovered chaplains that are bad at PPE.	Yes, IC does informal rounds, HH and PPE are also observed. Fingernail checks are done and quality managers do environment of care rounds
Does your facility provide feedback to personnel regarding their PPE use?	Yes, the JIT feedback is done by the MDRO coordinator.	Yes, discussed within management then to specific employees with routine feedback	Yes, IC provides feedback during informal rounds
Does your facility have supplies necessary for adherence to proper PPE use (e.g., gloves, gowns, masks) readily accessible in resident care areas (i.e., nursing units, therapy rooms)?	Yes. But the facility is limited in where they can store PPEs. The MDRO coordinator wants to create more PPE stations, since they are currently spread far apart.	Yes	Yes
**Environmental Cleaning & Disinfection**			

Does your facility have written cleaning/disinfection policies, which include routine and high-touch surface cleaning, and terminal cleaning and disinfection, within the following areas: NH resident rooms, NH resident rooms on contact precautions, Common areas within the NH, therapy gyms, Dialysis, Radiology, Radiation-Onc, Infusion clinic, and Outpatient Clinic?	Yes to all	Yes to all	Yes to all
Does your facility have cleaning/disinfection policies that include handling of equipment shared among residents (e.g., blood pressure cuffs, rehab therapy equipment, etc.)?	Yes, this facility has SOPs which are supposed to be done between patients by staff.	Yes, there is a role-playing video in the training and reusable equipment disinfection is reviewed.	Yes
Does your facility perform job-specific training and competency validation for personnel on cleaning and disinfection procedures at the time of employment?	Yes	Yes	Yes, RME coordinator does rounds and observations with feedback
Does your facility perform job-specific training and competency validation for personnel on cleaning and disinfection procedures annually?	Yes	Yes	Yes
Does your facility routinely audit (monitor and document) the quality of cleaning and disinfection procedures?	Yes, vericlean is used in patient rooms and the MDRO coordinator checks to see if they are done in the NH. Infection control gets a report of the facilities management results.	Yes, monitor cleaning of patient beds (UV marker), HH monitors. EMS has own procedures as well	Yes, RME coordinator performs audits with shared equipment while EMS staff does ATP monitoring on a routine basis. UV light is also used following terminal disinfection of patients with precautions
Does your facility provide feedback to personnel regarding the quality of cleaning and disinfection procedures?	Yes	Yes, monthly EMS meeting attended by an IP team member	Yes
Does your facility provide necessary supplies appropriate for cleaning and disinfection procedures (e.g., EPA-registered, including products labeled as effective against C. difficile and Norovirus)?	Yes	Yes	Yes
Is there a process for designing clean/ready-for-use equipment?	Yes, this facility uses different bag colors to indicate clean versus dirty. There are also tags on wheelchairs.	Yes, ready to use sheets, markers on the beds, IV poles with plastic signage, and clean/dirty utility rooms	Yes, clean supply rooms and make sure equipment is well-labeled
Who performs cleaning and how often is it performed in…			
Common areas within the NH (dining room, recreation area, or family rooms)	Housekeeping performs after each meal, and they mop the whole area during the afternoon and around midnight.	EMS, daily	EMS staff daily or more if needed
Therapy gyms	Staff cleans between each patient and housekeeping cleans in the evenings.	EMS, at least daily	EMS staff daily or more if needed
Dialysis	Staff cleans between patients and housekeeping cleans in the evenings. *for MDRO positive/precaution patients, housekeeping performs cleaning after.	EMS, at least daily	EMS staff daily or more if needed
Radiology	Staff cleans between patients and housekeeping cleans in the evenings. *for MDRO positive/precaution patients, housekeeping performs cleaning after.	EMS, at least daily	EMS staff daily or more if needed
Radiation	Staff cleans between patients and housekeeping cleans in the evenings.	EMS, at least daily	EMS staff daily or more if needed

	*for MDRO positive/precaution patients, housekeeping performs cleaning after.		
Infusion Clinic	Staff cleans between patients and housekeeping cleans in the evenings. *for MDRO positive/precaution patients, housekeeping performs cleaning after.	EMS, at least daily	EMS staff daily or more if needed
Outpatient clinics (e.g., eye clinic)	Weekly cleaning by housekeeping in general. Staff does clean in between patients. The dental office gets cleaned daily.	EMS, at least daily	EMS staff daily or more if needed

Data collected via one-hour interviews with each facility's infection prevention and control representative.

Abbreviations: ATP, adenosine triphosphate; CPRS, computerized patient record system; EMS, environmental management services; FTE, full-time equivalent; HH, hand hygiene; IC, infection control; IV, intravenous; JC, joint commission; MDRO, multidrug-resistant organism; NH, nursing home; PPE, personal protective equipment; QA, quality assurance; RME, reliability maintenance engineering; UV, ultraviolet.

Supplementary Table 5. Number of swabs positive out of swabs collected during in-room visits.

	No. Swabs Positive / No. Swabs Collected (%)				P-value		
**All MDROs**	**All Facilities**	**Facility A**	**Facility B**	**Facility C**	**A vs. B**	**A vs. C**	**B vs. C**
Nares	90/757 (11.9%)	29/392 (7.4%)	42/232 (18.1%)	19/133 (14.3%)			
Hand	151/757 (19.9%)	68/392 (17.3%)	55/232 (23.7%)	28/133 (21.1%)			
Groin	241/744 (32.4%)	116/391 (29.7%)	90/227 (39.6%)	35/126 (27.8%)			
Any Participant Body site[a]	482/2,258 (21.3%)	213/1,175 (18.1%)	187/691 (27.1%)	82/392 (20.9%)	0.04	0.592	0.342
Bed control	121/757 (16.0%)	50/393 (12.7%)	49/232 (21.1%)	22/132 (16.7%)			
Call button	96/758 (12.7%)	45/393 (11.5%)	37/232 (15.9%)	14/133 (10.5%)			
Table top	126/756 (16.7%)	47/393 (12.0%)	61/230 (26.5%)	18/133 (13.5%)			
TV remote/buttons	122/758 (16.1%)	54/393 (13.7%)	48/232 (20.7%)	20/133 (15.0%)			
Privacy curtain	133/744 (17.9%)	43/384 (11.2%)	76/232 (32.8%)	14/128 (10.9%)			
Bedrail	136/758 (17.9%)	55/393 (14.0%)	63/232 (27.2%)	18/133 (13.5%)			
Toilet seat	151/754 (20.0%)	74/392 (18.9%)	61/232 (26.3%)	16/130 (12.3%)			
Any Environmental site[b]	885/5,285 (16.7%)	368/2,741 (13.4%)	395/1,622 (24.4%)	122/922 (13.2%)	0.002	0.958	0.024
**MRSA**							
Nares	48/757 (6.3%)	16/392 (4.1%)	20/232 (8.6%)	12/133 (9.0%)			
Hand	49/757 (6.5%)	17/392 (4.3%)	20/232 (8.6%)	12/133 (9.0%)			
Groin	26/744 (3.5%)	6/391 (1.5%)	13/227 (5.7%)	7/126 (5.6%)			
Any Participant Body site[a]	123/2,258 (5.4%)	39/1,175 (3.3%)	53/691 (7.7%)	31/392 (7.9%)	0.059	0.126	0.954
Bed control	46/757 (6.1%)	14/393 (3.6%)	23/232 (9.9%)	9/132 (6.8%)			
Call button	37/758 (4.9%)	11/393 (2.8%)	16/232 (6.9%)	10/133 (7.5%)			
Table top	43/756 (5.7%)	12/393 (3.1%)	21/230 (9.1%)	10/133 (7.5%)			
TV remote/buttons	43/758 (5.7%)	15/393 (3.8%)	17/232 (7.3%)	11/133 (8.3%)			
Privacy curtain	29/744 (3.9%)	8/384 (2.1%)	15/232 (6.5%)	6/128 (4.7%)			
Bedrail	40/758 (5.3%)	13/393 (3.3%)	18/232 (7.8%)	9/133 (6.8%)			
Toilet seat	27/754 (3.6%)	7/392 (1.8%)	14/232 (6.0%)	6/130 (4.6%)			
Any Environmental site[b]	265/5,285 (5.0%)	80/2,741 (2.9%)	124/1,622 (7.6%)	61/922 (6.6%)	0.035	0.133	0.777
**VRE**							
Nares	28/757 (3.7%)	9/392 (2.3%)	16/232 (6.9%)	3/133 (2.3%)			
Hand	107/757 (14.1%)	53/392 (13.5%)	40/232 (17.2%)	14/133 (10.5%)			
Groin	173/744 (23.3%)	92/391 (23.5%)	59/227 (26.0%)	22/126 (17.5%)			
Any Participant Body site[a]	308/2,258 (13.6%)	154/1,175 (13.1%)	115/691 (16.6%)	39/392 (9.9%)	0.033	0.16	0.181
Bed control	82/757 (10.8%)	38/393 (9.7%)	30/232 (12.9%)	14/132 (10.6%)			
Call button	64/758 (8.4%)	33/393 (8.4%)	28/232 (12.1%)	3/133 (2.3%)			

Table top	86/756 (11.4%)	35/393 (8.9%)	43/230 (18.7%)	8/133 (6.0%)			
TV remote/buttons	84/758 (11.1%)	41/393 (10.4%)	35/232 (15.1%)	8/133 (6.0%)			
Privacy curtain	100/744 (13.4%)	34/384 (8.9%)	57/232 (24.6%)	9/128 (7.0%)			
Bedrail	95/758 (12.5%)	42/393 (10.7%)	45/232 (19.4%)	8/133 (6.0%)			
Toilet seat	115/754 (15.3%)	64/392 (16.3%)	43/232 (18.5%)	8/130 (6.2%)			
Any Environmental site[b]	626/5,285 (11.8%)	287/2,741 (10.5%)	281/1,622 (17.3%)	58/922 (6.3%)	0.354	0.441	0.007
**RGNB**							
Nares	19/757 (2.5%)	4/392 (1.0%)	11/232 (4.7%)	4/133 (3.0%)			
Hand	13/757 (1.7%)	4/392 (1.0%)	4/232 (1.7%)	5/133 (3.8%)			
Groin	85/744 (11.4%)	35/391 (9.0%)	34/227 (15.0%)	16/126 (12.7%)			
Any Participant Body site[a]	117/2,258 (5.2%)	43/1,175 (3.7%)	49/691 (7.1%)	25/392 (6.4%)	0.038	0.133	0.772
Bed control	8/757 (1.1%)	1/393 (0.3%)	6/232 (2.6%)	1/132 (0.8%)			
Call button	4/758 (0.5%)	2/393 (0.5%)	0/232	2/133 (1.5%)			
Table top	12/756 (1.6%)	5/393 (1.3%)	6/230 (2.6%)	1/133 (0.8%)			
TV remote/buttons	4/758 (0.5%)	1/393 (0.3%)	1/232 (0.4%)	2/133 (1.5%)			
Privacy curtain	10/744 (1.3%)	3/384 (0.8%)	7/232 (3.0%)	0/128 (0)			
Bedrail	13/758 (1.7%)	1/393 (0.3%)	8/232 (3.4%)	4/133 (3.0%)			
Toilet seat	20/754 (2.7%)	5/392 (1.3%)	13/232 (5.6%)	2/130 (1.5%)			
Any Environmental site[b]	71/5,285 (1.3%)	18/2,741 (0.7%)	41/1,622 (2.5%)	12/922 (1.3%)	0.002	0.31	0.327

Two-sided p-values for comparing odds of MDRO contamination or colonization across facilities are based on logistic regression and are adjusted for participant-level correlation in outcomes.

Abbreviations: MDRO, multidrug-resistant organism; MRSA, methicillin-resistant *Staphylococcus aureus*; RGNB, resistant gram-negative bacteria; VRE, vancomycin-resistant enterococci

[a]Number of participants colonized at any of the three body sites sampled (nares, hand, or groin).
[b]Number of participants with environmental contamination at any of the seven environmental sites sampled (bed control, call button, table top, TV remote, privacy curtain, bedrail, or toilet seat).

Supplementary Table 6. Number of swabs positive out of swabs collected during interactive visits.

	No. Swabs Positive / No. Swabs Collected (%)				P-value		
**All MDROs**	**All Facilities**	**Facility A**	**Facility B**	**Facility C**	**A vs. B**	**A vs. C**	**B vs. C**
Participant Hands	130/920 (14.1%)	100/656 (15.2%)	24/111 (21.6%)	6/153 (3.9%)	0.286	0.023	0.005
HCP Hands	18/314 (5.7%)	10/188 (5.3%)	5/86 (5.8%)	3/40 (7.5%)	0.863	0.589	0.714
All Surfaces/Equipment	83/1,682 (4.9%)	48/1,142 (4.2%)	27/283 (9.5%)	8/257 (3.1%)	0.027	0.615	0.072
Top 12 Surfaces							
Walker	11/220 (5.0%)	9/122 (7.4%)	1/31 (3.2%)	1/67 (1.5%)			
Wheelchair	2/60 (3.3%)	0/41 (0)	0/3 (0)	2/16 (12.5%)			
Pulse Ox	2/138 (1.4%)	1/123 (0.8%)	1/11 (9.1%)	0/4 (0)			
Stationary bike	3/86 (3.5%)	2/60 (3.3%)	1/20 (5.0%)	0/6 (0)			
Table top	5/115 (4.3%)	4/73 (5.5%)	1/38 (2.6%)	0/4 (0)			
Chair	6/57 (10.5%)	3/44 (6.8%)	3/12 (25.0%)	0/1 (0)			
Arm bike	0/59 (0)	0/51 (0)	0/4 (0)	0/4 (0)			
Weights	1/64 (1.6%)	1/60 (1.7%)	-	0/4 (0)			
Exercise band	2/21 (9.5%)	2/9 (22.2%)	0/6 (0)	0/6 (0)			
Food tray	2/49 (4.1%)	0/18 (0)	2/31 (6.5%)	-			
Cane	1/20 (5.0%)	1/16 (6.3%)	-	0/4 (0)			
Bedrail	0/9 (0)	0/2 (0)	-	0/7 (0)			
**MRSA**							
Participant Hands	36/920 (3.9%)	18/656 (2.7%)	13/111 (11.7%)	5/153 (3.3%)	0.007	0.826	0.082
HCP Hands	4/314 (1.3%)	1/188 (0.5%)	2/86 (2.3%)	1/40 (2.5%)	0.233	0.271	0.953
All Surfaces/Equipment	25/1,682 (1.5%)	10/1,142 (0.9%)	9/283 (3.2%)	6/257 (2.3%)	0.011	0.209	0.699
Top 12 Surfaces							
Walker	2/220 (0.9%)	1/122 (0.8%)	0/31 (0)	1/67 (1.5%)			
Wheelchair	2/60 (3.3%)	0/41 (0)	0/3 (0)	2/16 (12.5%)			
Pulse Ox	2/138 (1.4%)	1/123 (0.8%)	1/11 (9.1%)	0/4 (0)			
Stationary bike	0/86 (0)	0/60 (0)	0/20 (0)	0/6 (0)			
Table top	1/115 (0.9%)	0/73 (0)	1/38 (2.6%)	0/4 (0)			
Chair	0/57 (0)	0/44 (0)	0/12 (0)	0/1 (0)			
Arm bike	0/59 (0)	0/51 (0)	0/4 (0)	0/4 (0)			
Weights	0/64 (0)	0/60 (0)	-	0/4 (0)			
Exercise band	1/21 (4.8%)	1/9 (11.1%)	0/6 (0)	0/6 (0)			
Food tray	2/49 (4.1%)	0/18 (0)	2/31 (6.5%)	-			
Cane	0/20 (0)	0/16 (0)	-	0/4 (0)			

Bedrail	0/9 (0)	0/2 (0)	-	0/7 (0)			
**VRE**							
Participant Hands	93/920 (10.1%)	80/656 (12.2%)	12/111 (10.8%)	1/153 (0.7%)	0.816	0.004	0.01
HCP Hands	14/314 (4.5%)	9/188 (4.8%)	3/86 (3.5%)	2/40 (5.0%)	0.607	0.955	0.681
All Surfaces/Equipment	57/1,682 (3.4%)	37/1,142 (3.2%)	20/283 (7.1%)	0/257 (0)	0.092	-	-
Top 12 Surfaces							
Walker	10/220 (4.5%)	9/122 (7.4%)	1/31 (3.2%)	0/67 (0)			
Wheelchair	0/60 (0)	0/41 (0)	0/3 (0)	0/16 (0)			
Pulse Ox	0/138 (0)	0/123 (0)	0/11 (0)	0/4 (0)			
Stationary bike	3/86 (3.5%)	2/60 (3.3%)	1/20 (5.0%)	0/6 (0)			
Table top	4/115 (3.5%)	4/73 (5.5%)	0/38 (0)	0/4 (0)			
Chair	6/57 (10.5%)	3/44 (6.8%)	3/12 (25.0%)	0/1 (0)			
Arm bike	0/59 (0)	0/51 (0)	0/4 (0)	0/4 (0)			
Weights	1/64 (1.6%)	1/60 (1.7%)	-	0/4 (0)			
Exercise band	1/21 (4.8%)	1/9 (11.1%)	0/6 (0)	0/6 (0)			
Food tray	1/49 (2.0%)	0/18 (0)	1/31 (3.2%)	-			
Cane	1/20 (5.0%)	1/16 (0.6%)	-	0/4 (0)			
Bedrail	0/9 (0)	0/2 (0)	-	0/7 (0)			
**RGNB**							
Participant Hands	3/920 (0.3%)	3/656 (0.5%)	0/111 (0)	0/153 (0)	-	-	-
HCP Hands	2/314 (0.6%)	0/188 (0)	1/86 (1.2%)	1/40 (2.5%)	-	-	0.59
All Surfaces/Equipment	5/1,682 (0.3%)	2/1,142 (0.2%)	1/283 (0.4%)	2/257 (0.8%)	0.566	0.113	0.566
Top 12 Surfaces							
Walker	0/220 (0)	0/122 (0)	0/31 (0)	0/67 (0)			
Wheelchair	0/60 (0)	0/41 (0)	0/3 (0)	0/16 (0)			
Pulse Ox	0/138 (0)	0/123 (0)	0/11 (0)	0/4 (0)			
Stationary bike	0/86 (0)	0/60 (0)	0/20 (0)	0/6 (0)			
Table top	0/115 (0)	0/73 (0)	0/38 (0)	0/4 (0)			
Chair	0/57 (0)	0/44 (0)	0/12 (0)	0/1 (0)			
Arm bike	0/59 (0)	0/51 (0)	0/4 (0)	0/4 (0)			
Weights	0/64 (0)	0/60 (0)	-	0/4 (0)			
Exercise band	0/21 (0)	0/9 (0)	0/6 (0)	0/6 (0)			
Food tray	0/49 (0)	0/18 (0)	0/31 (0)	-			
Cane	0/20 (0)	0/16 (0)	-	0/4 (0)			
Bedrail	0/9 (0)	0/2 (0)	-	0/7 (0)			

Two-sided p-values for comparing odds of MDRO contamination or colonization across facilities are based on logistic regression and are adjusted for participant-level correlation in outcomes.

Abbreviations: HCP, healthcare personnel; MDRO, multidrug-resistant organism; MRSA, methicillin-resistant *Staphylococcus aureus*; RGNB, resistant gram-negative bacteria; VRE, vancomycin-resistant enterococci

Supplementary Figures

Supplementary Figure 1. Resident enrollment

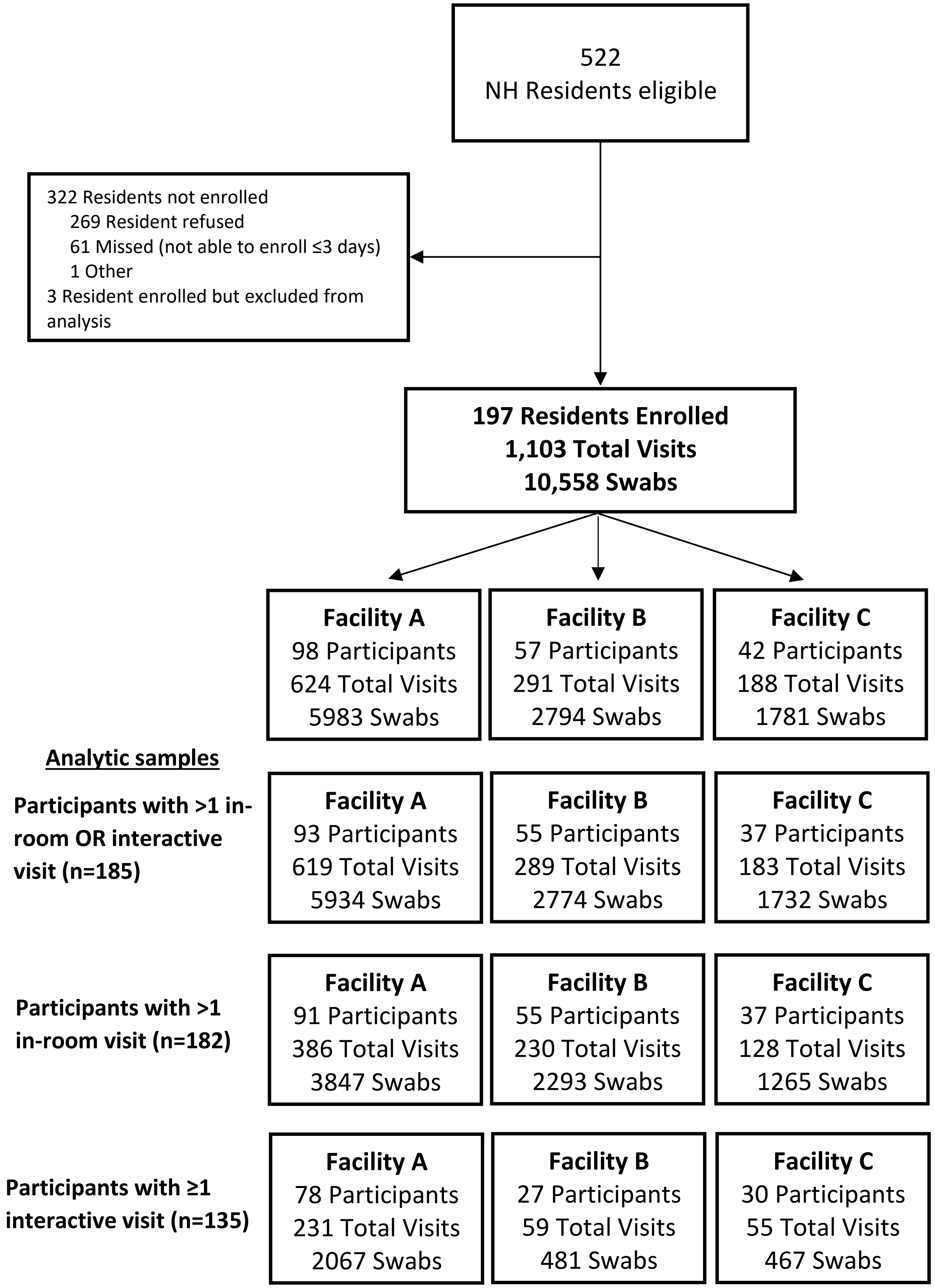

Supplementary Figure 1. Flow of data from eligible to final enrolled analytic sample. Among the 185 participants with at least one follow-up visit (in-room or interactive), 175 were seen on day seven, 139 on day 14, 110 on day 21, 91 on day 30, 28 on day 60, and 18 on day 90. Total days of follow-up for the sample of 185 participants was 6,442 days.

Supplementary Figure 2. Participant body sites colonized during in-room visits at facility A (a), B (b), and C (c)

a.

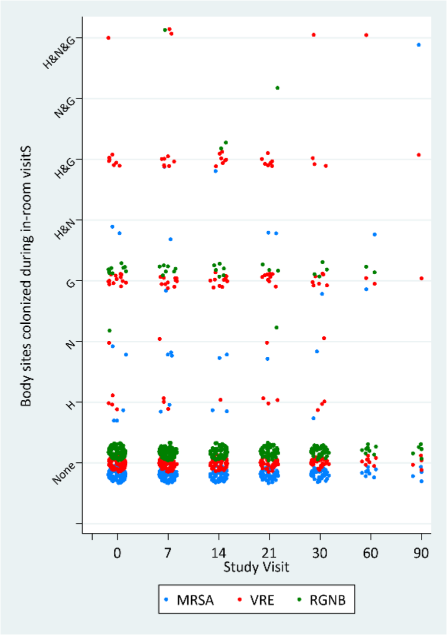

b.

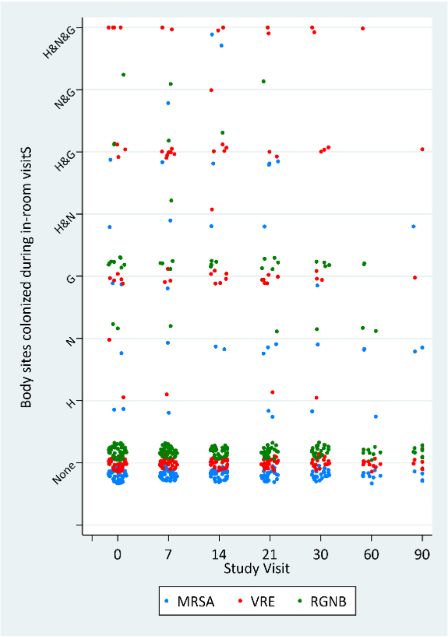

c.

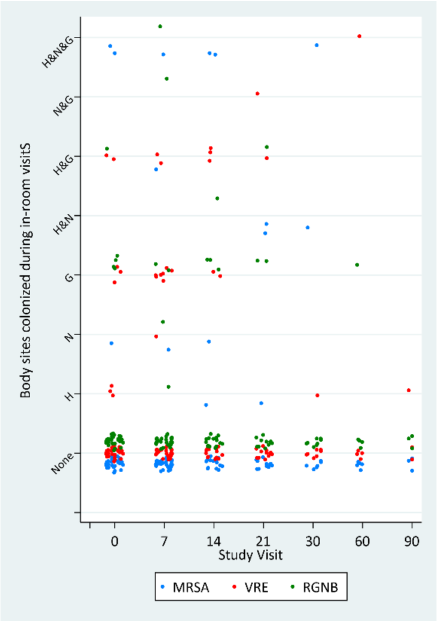

Supplementary Figure 2. Each participant is represented by a single dot. Blue dots indicate MRSA colonization; red dots indicate VRE colonization; green dots indicate R-GNB colonization. Study visits on the x-axis are numbered 0, 7, 14, 21, 30, 60, 90, as these visits were collected at study baseline (day 0), and then 7 days, 14 days, 21 days, 30 days, 60 days, and 90 days since baseline. Body sites colonized during in-room visits appears on the y-axis: colonization at zero body sites (none), a single body site only (N, nares; H, hand; or G, groin), as well as colonization with the same organism at multiple body sites (H&N, hand and nares; H&G, hand and groin; N&G, nares and groin; and H&N&G, hand and nares and groin) is included. Source data are provided for this figure. Abbreviations: MRSA, methicillin-resistant *Staphylococcus aureus*; RGNB, resistant gram-negative bacteria; VRE, vancomycin-resistant enterococci

Supplementary Figure 3. Participant body sites colonized during interactive visits at facility A (a), B (b), and C (c)

a.

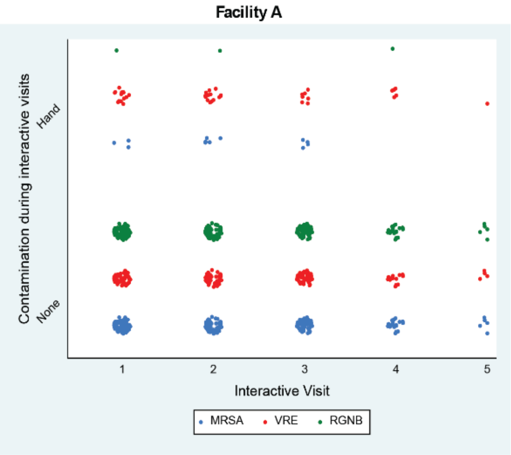

b.

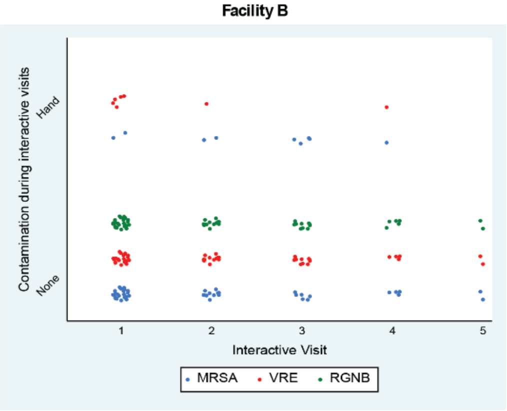

c.

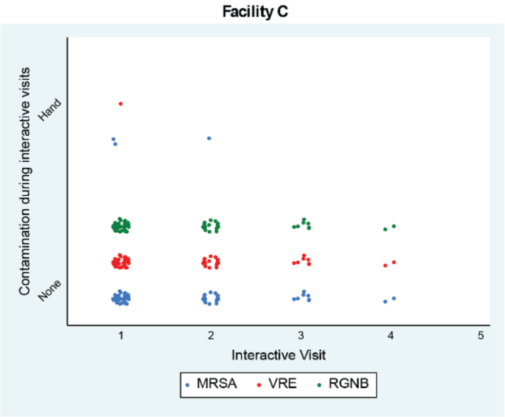

Supplementary Figure 3. Participant body sites colonized during interactive visits at facility A (a), B (b), and C (c). Each participant is represented by a single dot. Blue dots indicate MRSA colonization; red dots indicate VRE colonization; green dots indicate R-GNB colonization. Study visits on the x-axis are numbered 1-5. These visits were not conducted at a predetermined frequency, but rather at any point throughout a participant's stay in the study. On average, the first interactive visit occurred 9.3 days (SD 12.0) after study enrollment; the second interactive visit occurred 14.2 days (SD 14.7) after study enrollment; the third interactive visit occurred 20.3 days (SD 17.8) after study enrollment; the fourth interactive visit occurred 34.1 days (SD 26.0) after study enrollment; and the fifth interactive visit occurred 48.0 days (SD 31.2) after study enrollment. Source data are provided for this figure. Abbreviations: MRSA, methicillin-resistant *Staphylococcus aureus*; RGNB, resistant gram-negative bacteria; VRE, vancomycin-resistant enterococci

Supplementary Figure 4. Changes in MDRO colonization status from baseline to discharge at facilities A (a), B (b), and C (c)

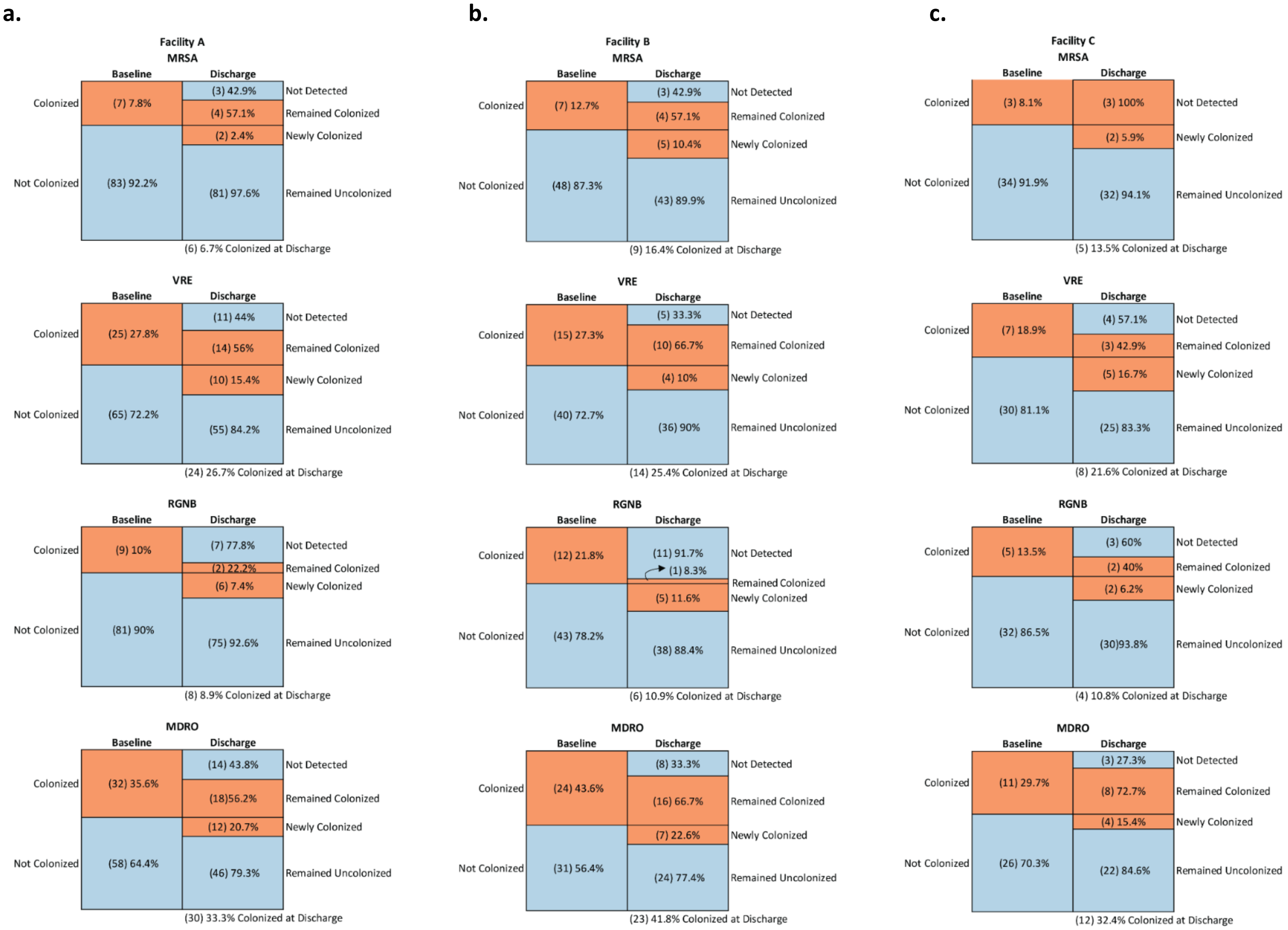

Supplementary Figure 4. Numbers in each box indicate the number of participants with a given colonization status at study baseline or discharge. For example, at Facility A, 25 participants who had multiple in-room visits were colonized with VRE at baseline. Of those, 14 (56.0%) remained colonized at discharge. The remaining 11 (44.0%) did not have detectable colonization and were clear at discharge. Alternatively, 65 participants who had multiple in-room visits were not colonized with VRE at baseline. Of those, 10 (15.4%) acquired VRE during their NH stay, while 55 (84.2%) remained not colonized with VRE. Source data are provided for this figure. Abbreviations: MDRO, multidrug-resistant organism; MRSA, methicillin-resistant *Staphylococcus aureus*; NH, nursing home; RGNB, resistant gram-negative bacilli; VRE, vancomycin-resistant enterococci. Source data are provided for this figure.

Supplementary Figure 5. Genomics support of microbiological linkages among two participants

a.

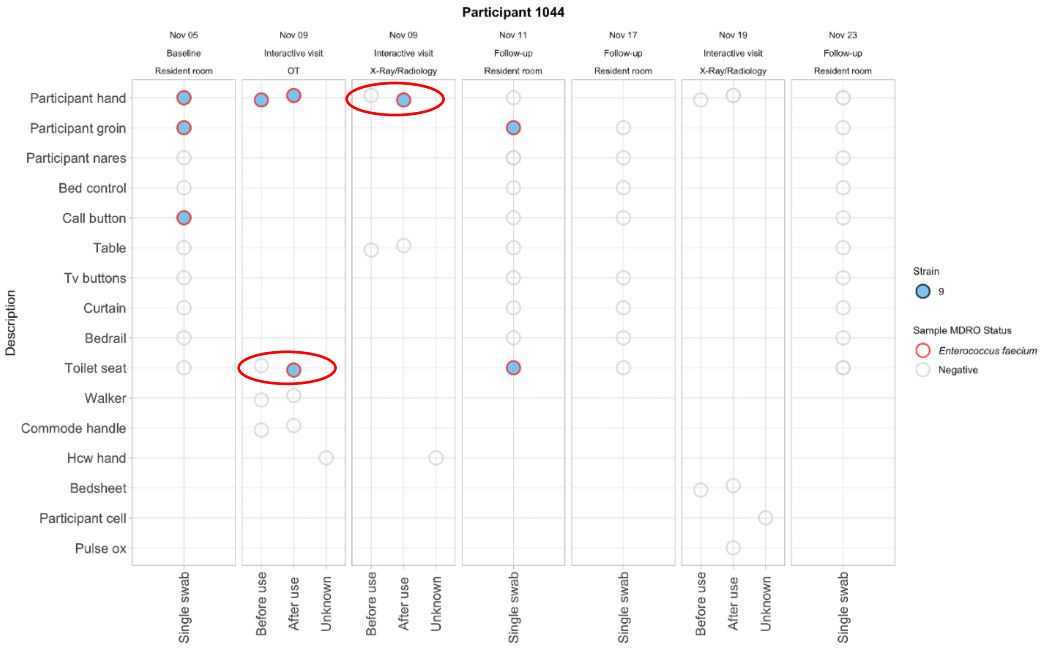

b.

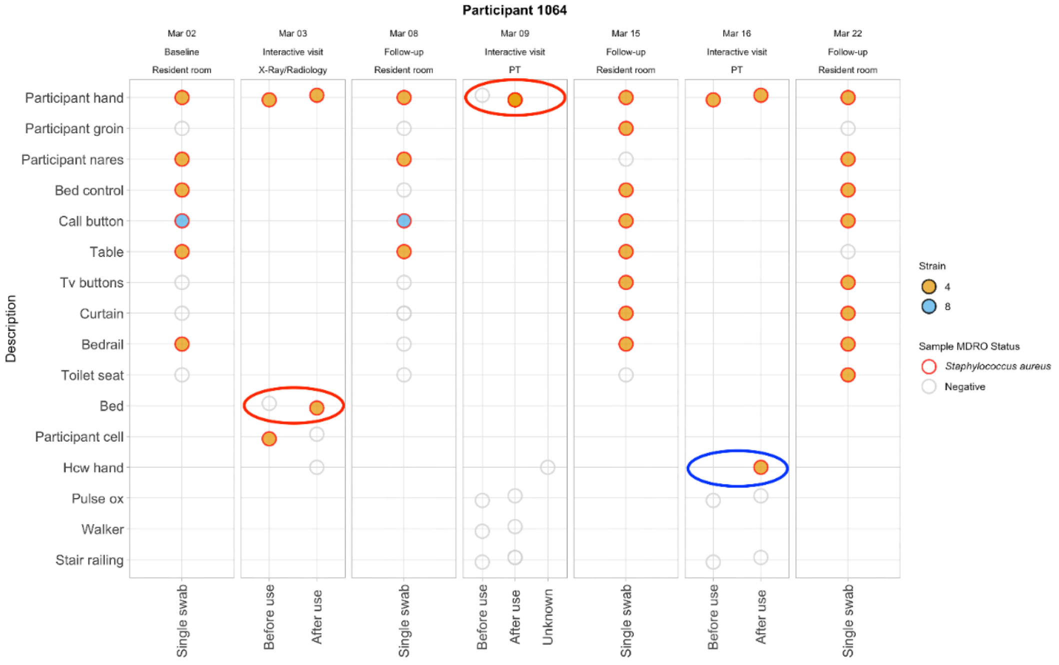

Supplementary Figure 5. Column headings indicate date, location, and type (in-room: baseline or follow-up; or interactive) of each study visit. Transmission events are identified with a red circle; single positive swabs not able to be assessed for transmission are identified with a blue circle. White circles outlined gray are samples collected and negative for any MDRO; colored circles outlined in red are samples collected and positive for a particular strain (listed in each legend). **a.** For participant 1044, VRE strain 9 was detected at multiple body sites and an environmental surface at study baseline. Transmission of VRE strain 9 occurred at an interactive visit to OT (Nov 9). The participant's hand was colonized with VRE throughout the OT session, and transmission of an identical VRE strain from the participant's hand (source) to the toilet seat was confirmed by genomic sequencing. At the second interactive visit that same day (Nov 9) to Radiology, the participant's hand is negative for any MDRO at the start of the session, but became positive for VRE strain 9 at the

end of the session. Within this interactive visit alone, it is not clear where that VRE comes from on the participant hand (unknown source); however, sequencing allows us to conclude that this participant was transiently colonized at the hand with VRE strain 9. **b.** For participant 1064, MRSA strain 4 was detected at multiple body sites and environmental surfaces at study baseline, as well as MRSA strain 8 at one environmental surface. Transmission of MRSA strain 4 occurred at an interactive visit to Radiology (X-ray) (March 3). The bed goes from negative to positive for MRSA strain 4 following participant use, and the participant's hand was colonized with that same strain at the start of the session, confirming it is the source of the transmission. At the second interactive visit to PT (March 9), the participant's hand is negative for any MDRO at the start of the session but becomes positive for MRSA strain 4 at the end of the session. Within this interactive visit alone, it is not clear where that MRSA comes from on the participant hand (unknown source); however, sequencing allows us to conclude that this participant was transiently colonized at the hand with MRSA strain 4. Abbreviations: MDRO, multidrug-resistant organism; OT, occupational therapy; PT, physical therapy; VRE, vancomycin-resistant enterococci.

Supplementary Figure 6. MRSA and VRE strain diversity in the overall participant population and between epidemiologically linked carriers.

a.

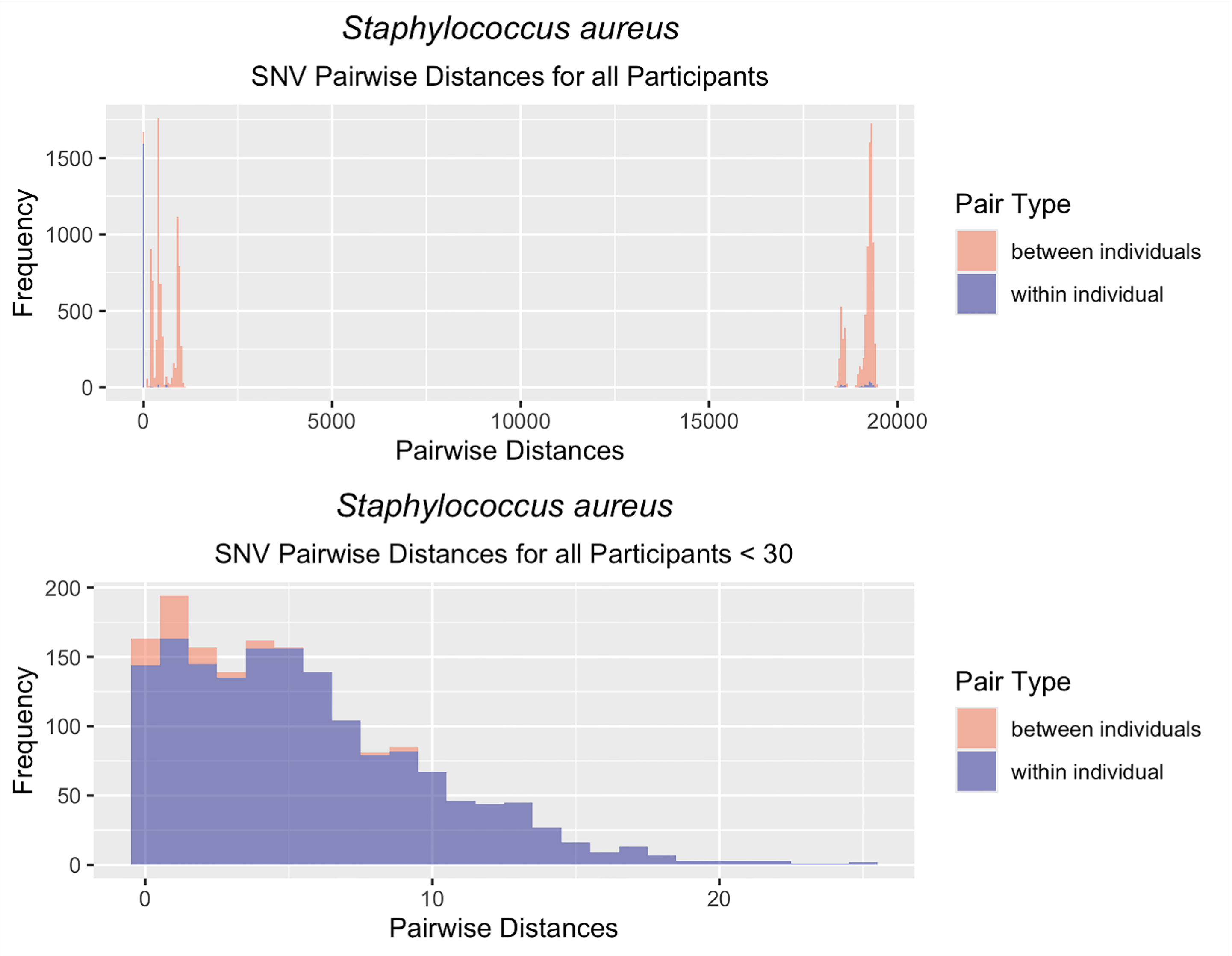

b.

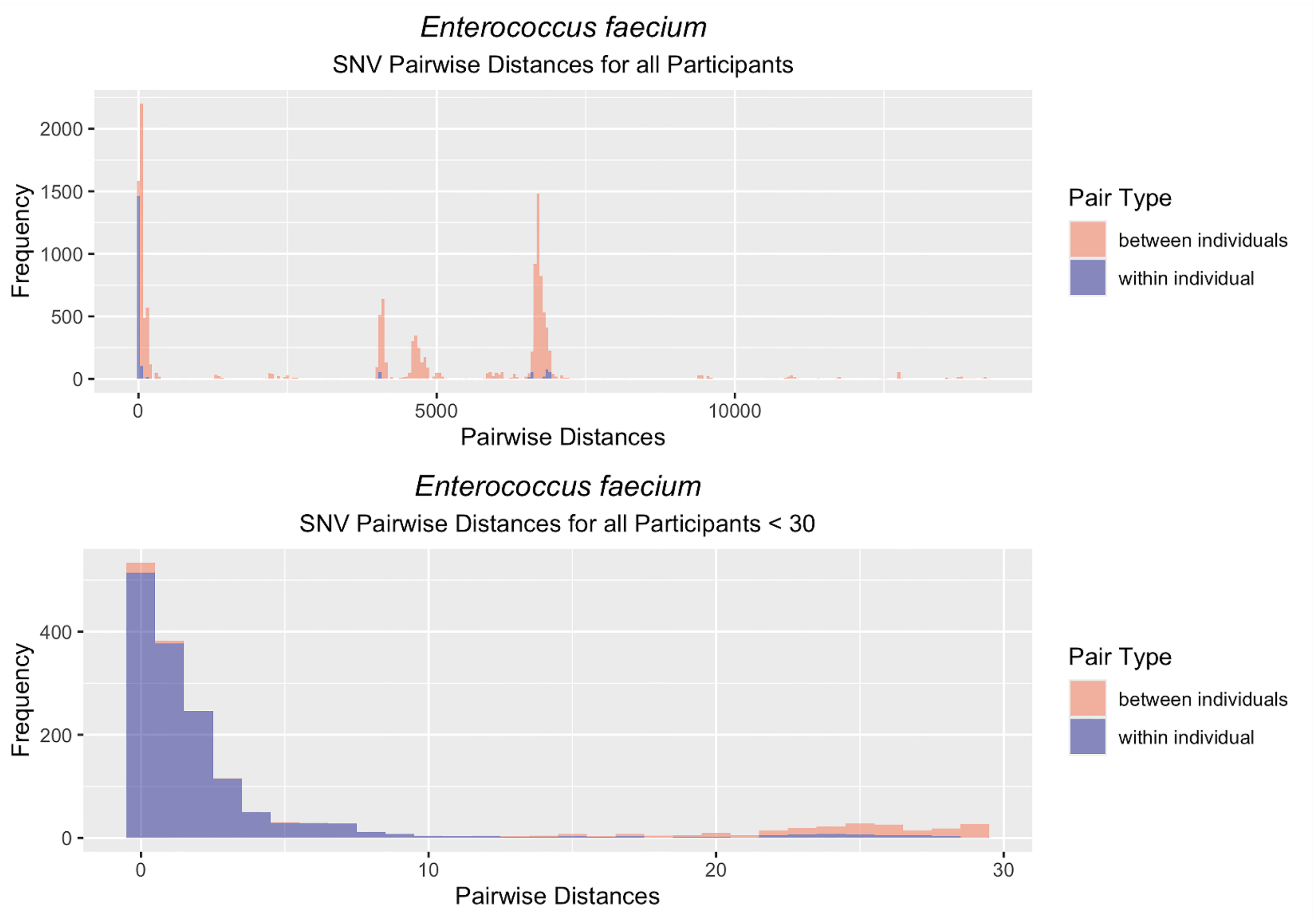
Enterococcus faecium
SNV Pairwise Distances for all Participants
Frequency
2000
1500
1000
500
0
0
5000
10000
Pairwise Distances
Pair Type
between individuals
within individual
Enterococcus faecium
SNV Pairwise Distances for all Participants < 30
Frequency
400
200
0
0
10
20
30
Pairwise Distances
Pair Type
between individuals
within individual

c.

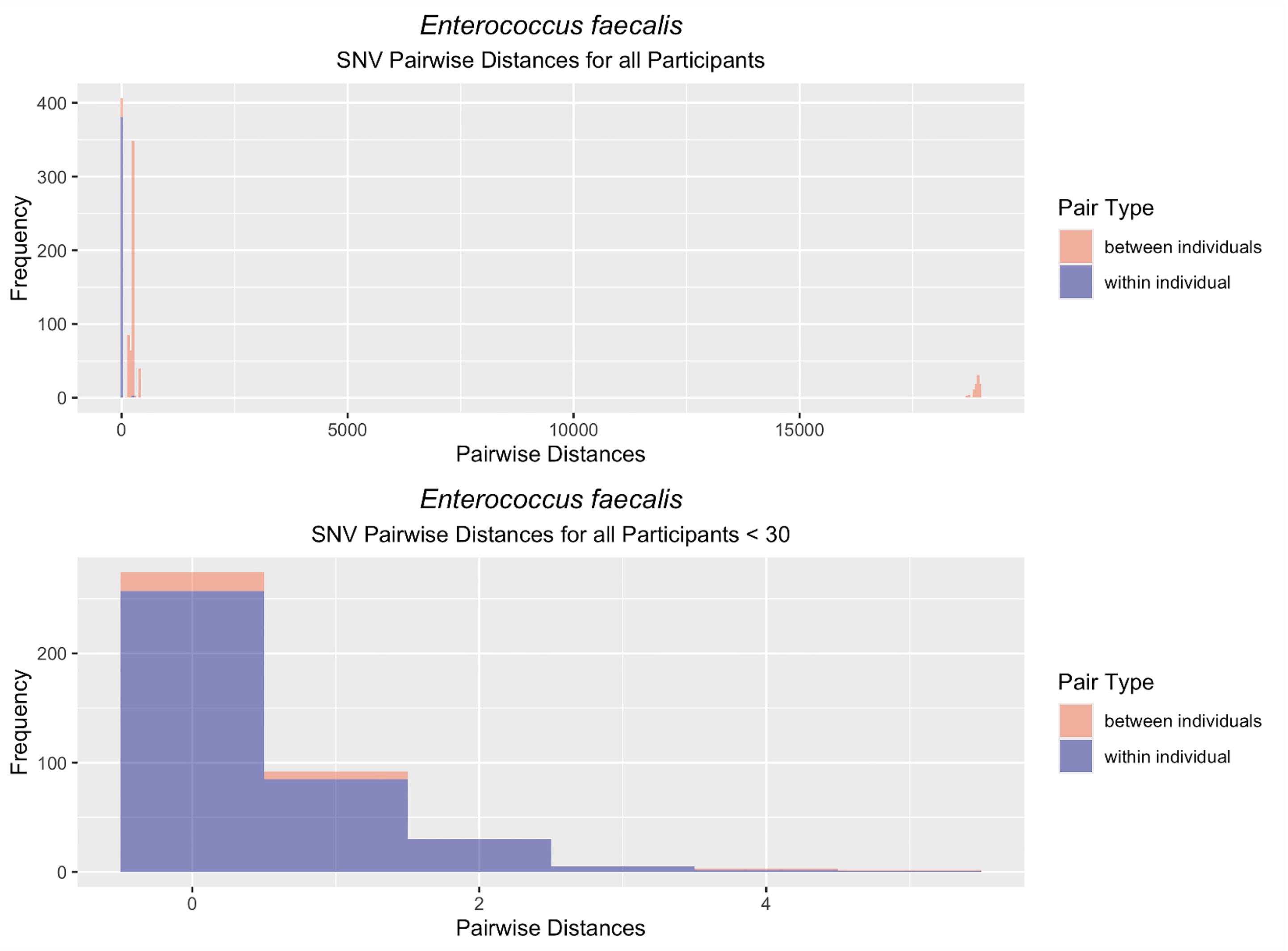

Supplementary Figure 6. MRSA and VRE strain diversity in the overall participant population and between epidemiologically linked carriers. **a.** Genomic distance between *Staphylococcus aureus* isolates between individuals (orange) compared to within individuals (blue). **b.** Genomic distance between *Enterococcus faecium* isolates between individuals (orange) compared to within individuals (blue). **c.** Genomic distance between *Enterococcus faecalis* isolates between individuals (orange) compared to within individuals (blue).

Supplementary Figure 7. Phylogenies for: a. Facility A *E. faecium*; b. Facility A *S. aureus*; c. Facility B *E. faecium*; d. Facility B *S. aureus*; e. Facility C *S. aureus*; and f. All *E. faecalis*

a.

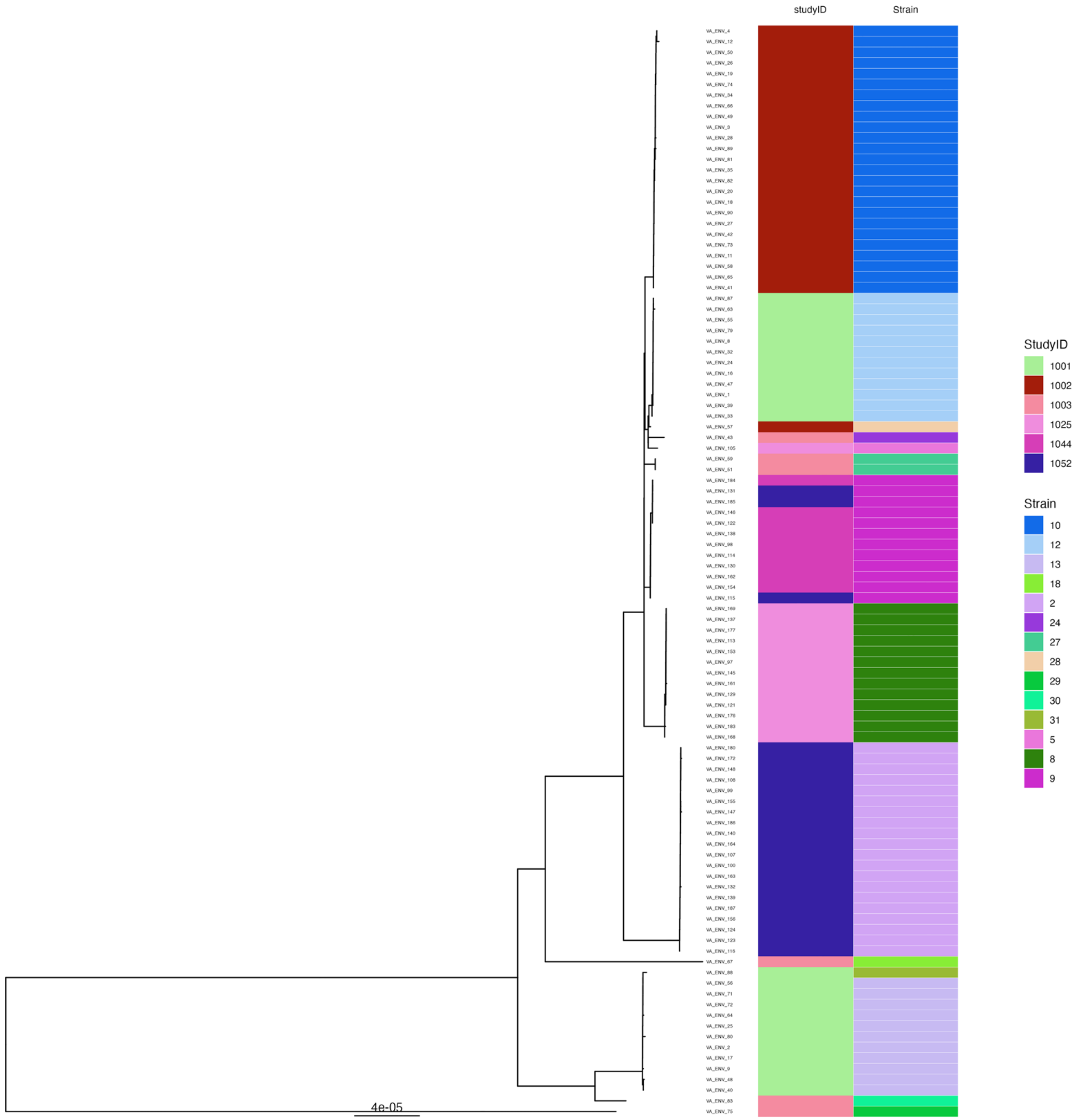

b.

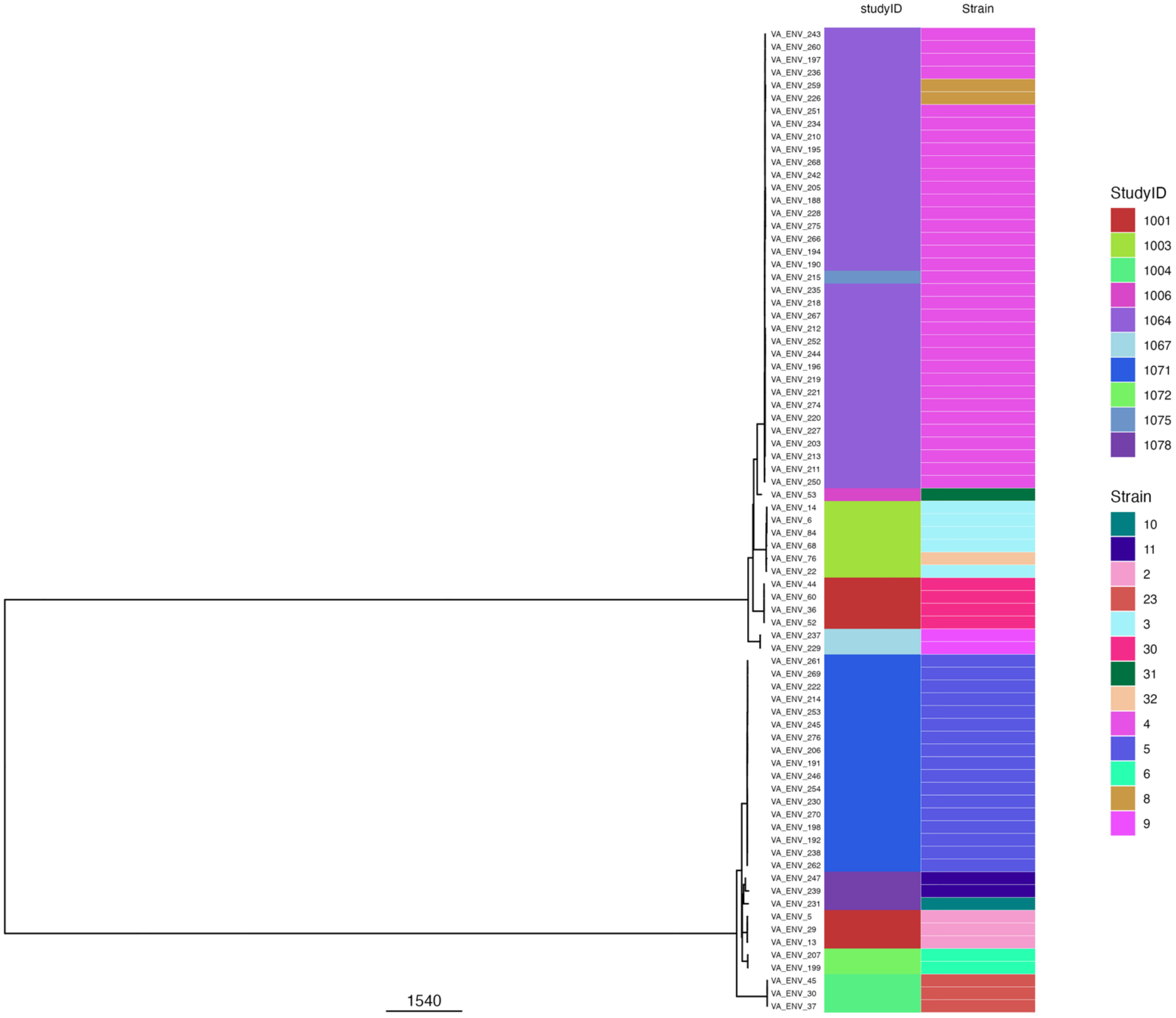
studyID
Strain
VA_ENV_243
VA_ENV_260
VA_ENV_197
VA_ENV_236
VA_ENV_259
VA_ENV_226
VA_ENV_251
VA_ENV_234
VA_ENV_210
VA_ENV_195
VA_ENV_268
VA_ENV_242
VA_ENV_205
VA_ENV_188
VA_ENV_228
VA_ENV_275
VA_ENV_266
VA_ENV_194
VA_ENV_190
VA_ENV_215
VA_ENV_235
VA_ENV_218
VA_ENV_267
VA_ENV_212
VA_ENV_252
VA_ENV_244
VA_ENV_196
VA_ENV_219
VA_ENV_221
VA_ENV_274
VA_ENV_220
VA_ENV_227
VA_ENV_203
VA_ENV_213
VA_ENV_211
VA_ENV_250
VA_ENV_53
VA_ENV_14
VA_ENV_6
VA_ENV_84
VA_ENV_68
VA_ENV_76
VA_ENV_22
VA_ENV_44
VA_ENV_60
VA_ENV_36
VA_ENV_52
VA_ENV_237
VA_ENV_229
VA_ENV_261
VA_ENV_269
VA_ENV_222
VA_ENV_214
VA_ENV_253
VA_ENV_245
VA_ENV_276
VA_ENV_206
VA_ENV_191
VA_ENV_246
VA_ENV_254
VA_ENV_230
VA_ENV_270
VA_ENV_198
VA_ENV_192
VA_ENV_238
VA_ENV_262
VA_ENV_247
VA_ENV_239
VA_ENV_231
VA_ENV_5
VA_ENV_29
VA_ENV_13
VA_ENV_207
VA_ENV_199
VA_ENV_45
VA_ENV_30
VA_ENV_37
1540
StudyID
1001
1003
1004
1006
1064
1067
1071
1072
1075
1078
Strain
10
11
2
23
3
30
31
32
4
5
6
8
9

c.

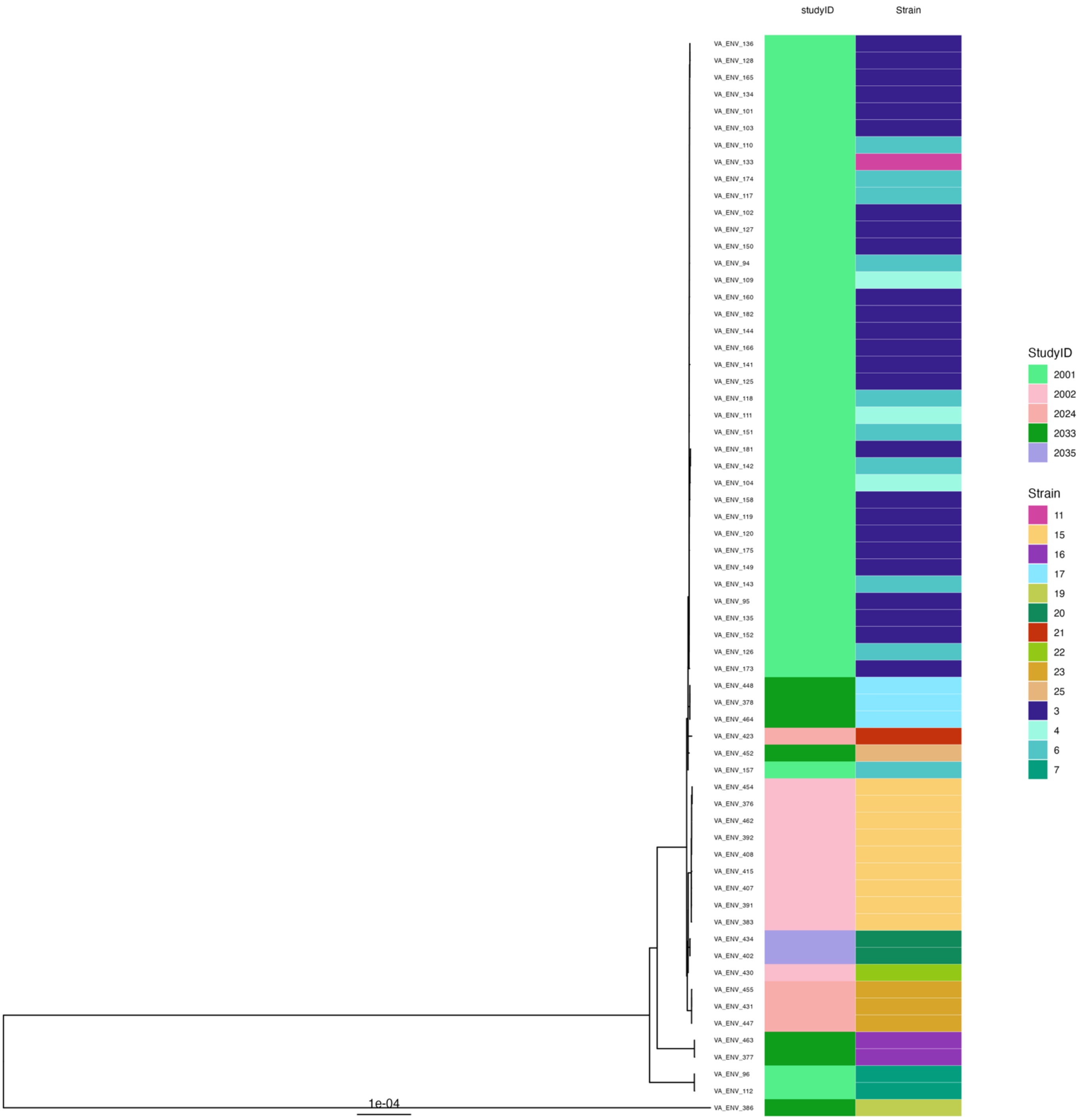
studyID
Strain
VA_ENV_136
VA_ENV_128
VA_ENV_165
VA_ENV_134
VA_ENV_101
VA_ENV_103
VA_ENV_110
VA_ENV_133
VA_ENV_174
VA_ENV_117
VA_ENV_102
VA_ENV_127
VA_ENV_150
VA_ENV_94
VA_ENV_109
VA_ENV_160
VA_ENV_182
VA_ENV_144
VA_ENV_166
VA_ENV_141
VA_ENV_125
VA_ENV_118
VA_ENV_111
VA_ENV_151
VA_ENV_181
VA_ENV_142
VA_ENV_104
VA_ENV_158
VA_ENV_119
VA_ENV_120
VA_ENV_175
VA_ENV_149
VA_ENV_143
VA_ENV_95
VA_ENV_135
VA_ENV_152
VA_ENV_126
VA_ENV_173
VA_ENV_448
VA_ENV_378
VA_ENV_464
VA_ENV_423
VA_ENV_452
VA_ENV_157
VA_ENV_454
VA_ENV_376
VA_ENV_462
VA_ENV_392
VA_ENV_408
VA_ENV_415
VA_ENV_407
VA_ENV_391
VA_ENV_383
VA_ENV_434
VA_ENV_402
VA_ENV_430
VA_ENV_455
VA_ENV_431
VA_ENV_447
VA_ENV_463
VA_ENV_377
VA_ENV_96
VA_ENV_112
VA_ENV_386
1e-04
StudyID
2001
2002
2024
2033
2035
Strain
11
15
16
17
19
20
21
22
23
25
3
4
6
7

d.

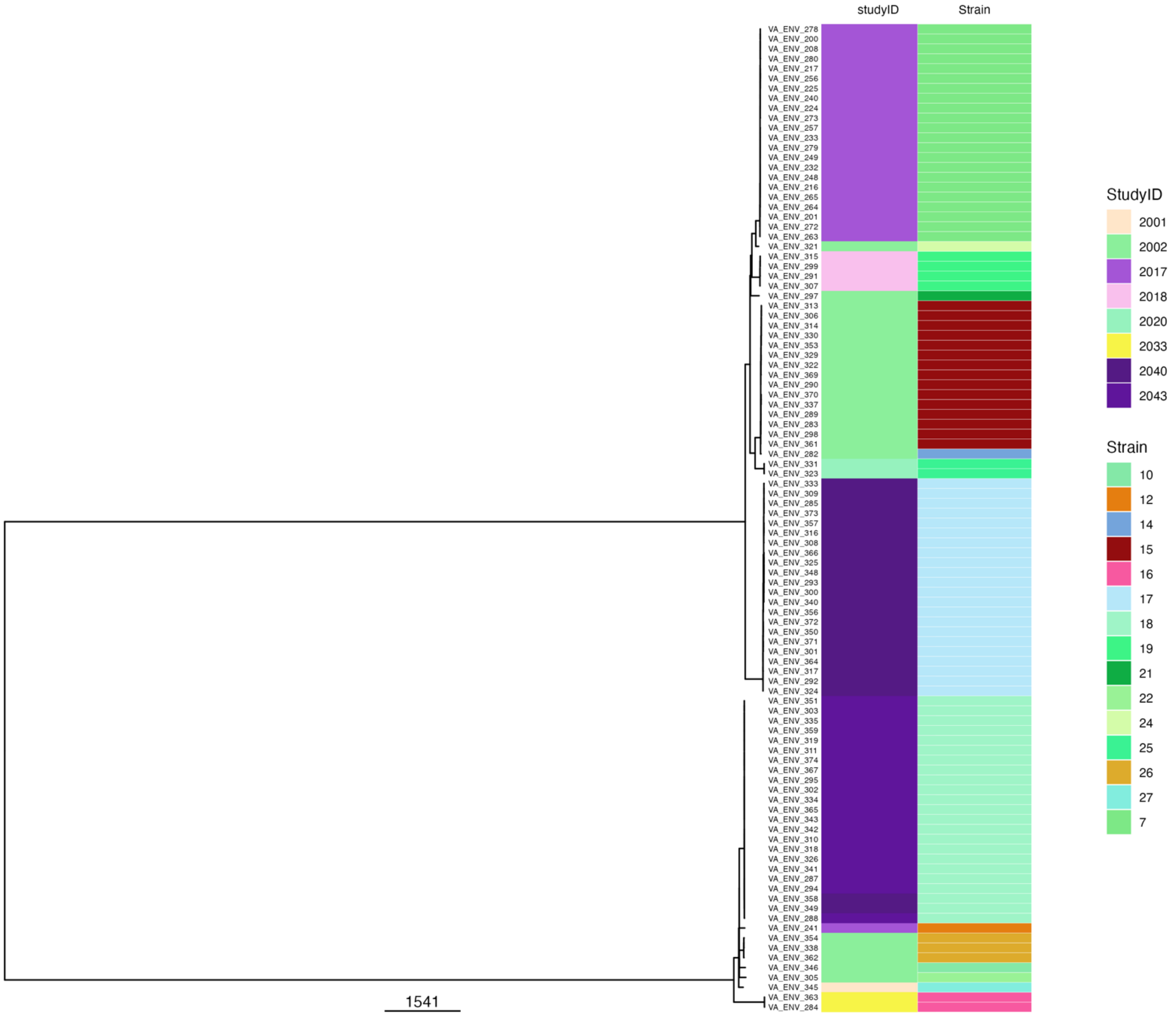

studyID
Strain
VA_ENV_278
VA_ENV_200
VA_ENV_208
VA_ENV_280
VA_ENV_217
VA_ENV_256
VA_ENV_225
VA_ENV_240
VA_ENV_224
VA_ENV_273
VA_ENV_257
VA_ENV_233
VA_ENV_279
VA_ENV_249
VA_ENV_232
VA_ENV_248
VA_ENV_216
VA_ENV_265
VA_ENV_264
VA_ENV_201
VA_ENV_272
VA_ENV_263
VA_ENV_321
VA_ENV_315
VA_ENV_299
VA_ENV_291
VA_ENV_307
VA_ENV_297
VA_ENV_313
VA_ENV_306
VA_ENV_314
VA_ENV_330
VA_ENV_353
VA_ENV_329
VA_ENV_322
VA_ENV_369
VA_ENV_290
VA_ENV_370
VA_ENV_337
VA_ENV_289
VA_ENV_283
VA_ENV_298
VA_ENV_361
VA_ENV_282
VA_ENV_331
VA_ENV_323
VA_ENV_333
VA_ENV_309
VA_ENV_285
VA_ENV_373
VA_ENV_357
VA_ENV_316
VA_ENV_308
VA_ENV_366
VA_ENV_325
VA_ENV_348
VA_ENV_293
VA_ENV_300
VA_ENV_340
VA_ENV_356
VA_ENV_372
VA_ENV_350
VA_ENV_371
VA_ENV_301
VA_ENV_364
VA_ENV_317
VA_ENV_292
VA_ENV_324
VA_ENV_351
VA_ENV_303
VA_ENV_335
VA_ENV_359
VA_ENV_319
VA_ENV_311
VA_ENV_374
VA_ENV_367
VA_ENV_295
VA_ENV_302
VA_ENV_334
VA_ENV_365
VA_ENV_343
VA_ENV_342
VA_ENV_310
VA_ENV_318
VA_ENV_326
VA_ENV_341
VA_ENV_287
VA_ENV_294
VA_ENV_358
VA_ENV_349
VA_ENV_288
VA_ENV_241
VA_ENV_354
VA_ENV_338
VA_ENV_362
VA_ENV_346
VA_ENV_305
VA_ENV_345
VA_ENV_363
VA_ENV_284
1541
StudyID
2001
2002
2017
2018
2020
2033
2040
2043
Strain
10
12
14
15
16
17
18
19
21
22
24
25
26
27
7

e.

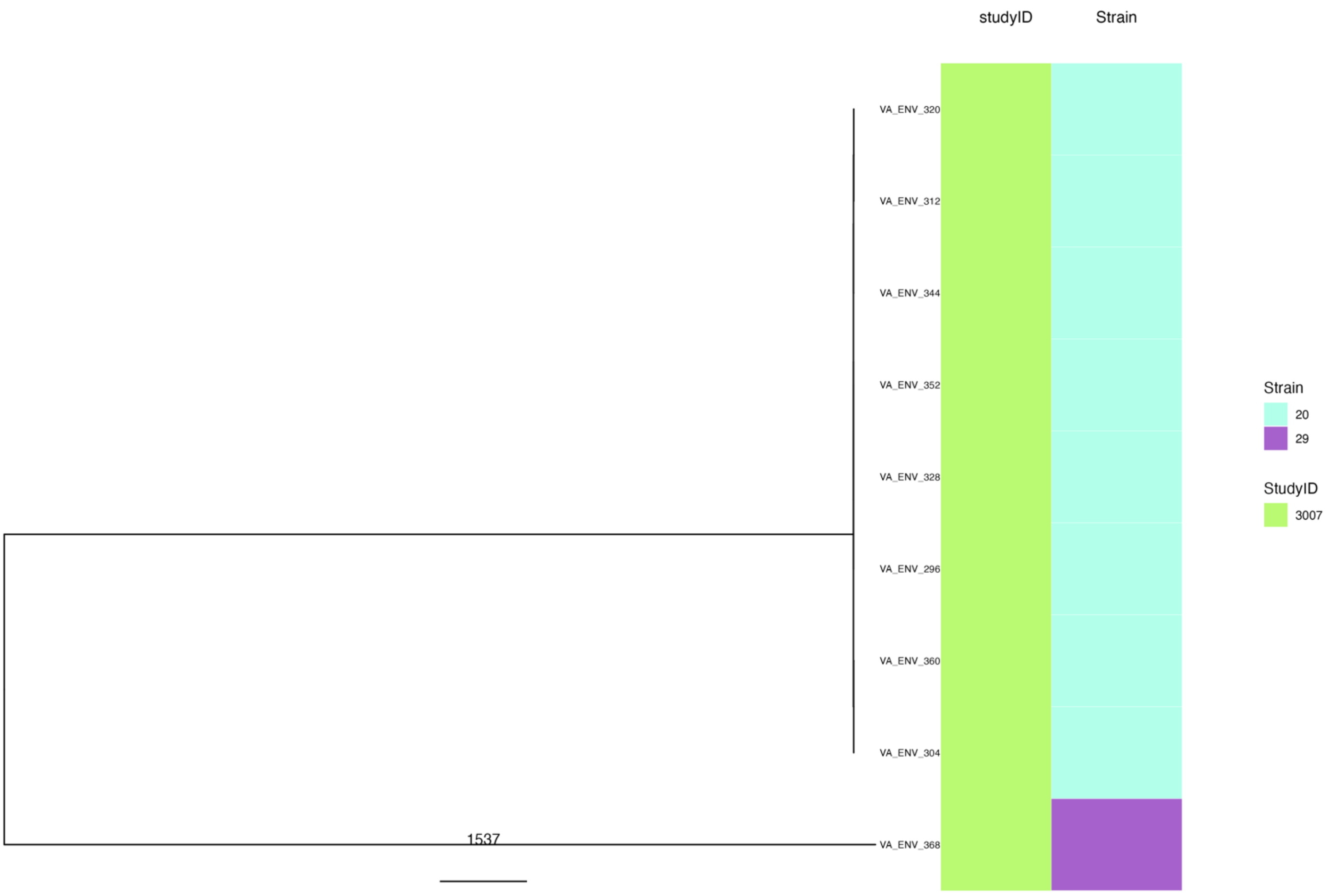
studyID
Strain
VA_ENV_320
VA_ENV_312
VA_ENV_344
VA_ENV_352
VA_ENV_328
VA_ENV_296
VA_ENV_360
VA_ENV_304
VA_ENV_368
1537
Strain
20
29
StudyID
3007

f.

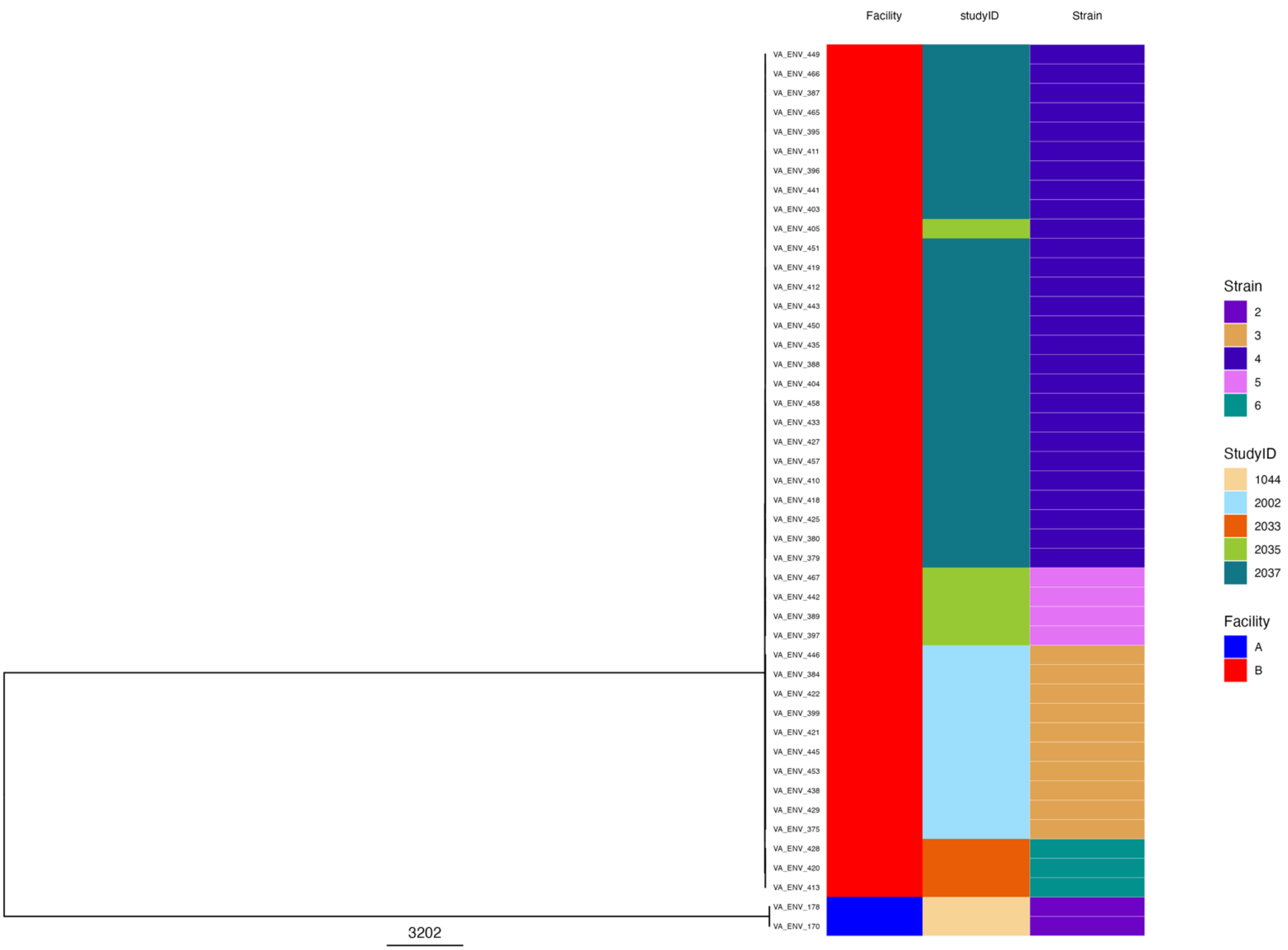

Supplementary Figure 7. Whole-genome maximum likelihood phylogenies of multidrug-resistant isolates from facilities A, B, and C were created using IQtree. The scale bar indicates substitutions per site. Adjacent heatmaps indicate the study ID of the participant from which an isolate was associated with (e.g. on the participant, in their room, during their visit) and the assigned strain groups using species-specific SNF cutoffs (see main text Methods and Supplemental Figure 6, 10 SNFs for VRE and 20 SNVs for MRSA). **a.** Facility A *E. faecium* phylogeny. **b.** Facility A *S. aureus* phylogeny. **c.** Facility B *E. faecium* phylogeny. **d.** Facility B *S. aureus* phylogeny. **e.** Facility C *S. aureus* phylogeny. **f.** All *E. faecalis* phylogeny
