# Supplementary material for: Epidemiology and transmission dynamics of multidrug-resistant organisms in nursing homes within the United States
Source: Nat Commun. 2025 Mar 13;16:2487. doi: 10.1038/s41467-025-57566-3 (PMC11906779; doi:10.1038/s41467-025-57566-3)
Supplement: Supplementary file 2 — Reporting Summary [file 41467_2025_57566_MOESM2_ESM.pdf]

nature portfolio

Corresponding author(s): Lona Mody, MD, MSc

Last updated by author(s): Feb 13, 2025

Reporting Summary

Nature Portfolio wishes to improve the reproducibility of the work that we publish. This form provides structure for consistency and transparency in reporting. For further information on Nature Portfolio policies, see our Editorial Policies and the Editorial Policy Checklist.

Statistics

For all statistical analyses, confirm that the following items are present in the figure legend, table legend, main text, or Methods section.

n/a	Confirmed	
☐	☒	The exact sample size (*n*) for each experimental group/condition, given as a discrete number and unit of measurement
☐	☒	A statement on whether measurements were taken from distinct samples or whether the same sample was measured repeatedly
☐	☒	The statistical test(s) used AND whether they are one- or two-sided<br>*Only common tests should be described solely by name; describe more complex techniques in the Methods section.*
☐	☒	A description of all covariates tested
☒	☐	A description of any assumptions or corrections, such as tests of normality and adjustment for multiple comparisons
☐	☒	A full description of the statistical parameters including central tendency (e.g. means) or other basic estimates (e.g. regression coefficient) AND variation (e.g. standard deviation) or associated estimates of uncertainty (e.g. confidence intervals)
☐	☒	For null hypothesis testing, the test statistic (e.g. *F*, *t*, *r*) with confidence intervals, effect sizes, degrees of freedom and *P* value noted<br>*Give P values as exact values whenever suitable.*
☒	☐	For Bayesian analysis, information on the choice of priors and Markov chain Monte Carlo settings
☐	☒	For hierarchical and complex designs, identification of the appropriate level for tests and full reporting of outcomes
☐	☒	Estimates of effect sizes (e.g. Cohen's *d*, Pearson's *r*), indicating how they were calculated

Our web collection on statistics for biologists contains articles on many of the points above.

Software and code

Policy information about availability of computer code

Data collection	Clinical data was collected from each participant's VA electronic medical record (CPRS) and entered, checked, and stored in Microsoft Access by trained research staff. Microbiological results of all swab specimens collected in the study were entered, checked, and stored in Microsoft Access by trained research staff.
Data analysis	All statistical analyses were performed using Stata 17 software (StataCorp. 2021. Stata Statistical Software: Release 17. College Station, TX: StataCorp LLC) and Mplus software version 8.8 (Muthén & Muthén).<br><br>Genomic libraries were prepared with the QIASeq FX DNA library prep kit and sequenced at the University of Michigan Advanced Genomics Core on an Illumina NovaSeq 6000, with 150-bp paired-end reads. Raw sequencing reads were trimmed using Trimmomatic v.0.39 to remove adapters and low-quality bases. Trimmed high-quality reads were assembled using Spades v.3.15.3, annotated using Prokka v.1.14.5, and had MLST assigned using the MLST tool. Trimmed sequencing reads were then mapped to species-specific reference genomes (E. faecium - Aus0004/GenBank CP003351.1, E. faecalis - V583/GenBank NC_004668.1, MRSA - USA300_TCH1516/ NC_010079.1) using the Burrows–Wheeler Aligner-MEM v.0.7.17 and variants were called and filtered using Samtools v.1.11. Variants were filtered from raw results using GATK's VariantFiltration (QUAL, >100; MQ, >50; >=10 reads supporting variant; and FQ, <0.025). In addition, a custom python script was used to filter out single- nucleotide variants that were: (i) <5 base pairs (bp) in proximity to indels that were identified by GATK HaplotypeCaller, (ii) in a phage region identified by Phaster or (iii) they resided in tandem repeats of length greater than 20bp as determined using the exact-tandem program in MUMmer.

For manuscripts utilizing custom algorithms or software that are central to the research but not yet described in published literature, software must be made available to editors and reviewers. We strongly encourage code deposition in a community repository (e.g. GitHub). See the Nature Portfolio guidelines for submitting code & software for further information.

Data

Policy information about availability of data

All manuscripts must include a data availability statement. This statement should provide the following information, where applicable:

- Accession codes, unique identifiers, or web links for publicly available datasets
- A description of any restrictions on data availability
- For clinical datasets or third party data, please ensure that the statement adheres to our policy

Sequence data that support the findings of this study (corresponding to Figure 4 and Supplementary Figures 5, 6, & 7) have been deposited in the National Center for Biotechnology Information (NCBI) BioSample database, with Bioproject number PRJNA1204323 (https://www.ncbi.nlm.nih.gov/bioproject/?term=PRJNA1204323). The source data underlying Figures and Supplementary Figures are provided as Source Data File. Additional details on datasets (such as aggregated data) and protocols that support findings of this study will be made available by the corresponding author (Lona Mody, lonamody@med.umich.edu) upon reasonable written request, in accordance with our VA-funded data management and access plan and with appropriate permissions from the VA Central Review Board. The authors will give feedback within 30 days.
This publication made use of the PubMLST website (https://pubmlst.org/) developed by Keith Jolley (Jolley & Maiden 2010, BMC Bioinformatics, 11:595) and sited at the University of Oxford. The development of that website was funded by the Wellcome Trust.

Research involving human participants, their data, or biological material

Policy information about studies with human participants or human data. See also policy information about sex, gender (identity/presentation), and sexual orientation and race, ethnicity and racism.

Reporting on sex and gender	Participant's self-reported sex was collected from their electronic medical record by trained research staff. Of the 197 participants in our study, 190 (96%) identified as male (98% at facility A; 100% at facility B; 88% at facility C). No sex-based analyses were performed, as our study population (Veterans) is predominantly male.
Reporting on race, ethnicity, or other socially relevant groupings	Participant's self-reported race was collected from their electronic medical record by trained research staff. Of the 197 participants in our study, 122 (64.2%) identified as White/Caucasian, 62 (32.6%) identified as Black/African American, and 6 (3.2%) identified as a race besides White or Black. We did observe facility-level differences in race— the percent of participants identifying as White at Facilities A, B, and C were 81%, 59%, and 33%, respectively; the percent of participants identifying as Black at Facilities A, B, and C were 15%, 38%, and 68%, respectively.
Population characteristics	Across all three cohorts, the median length of follow-up in the study was 28.0 days (IQR=13-41). The median length of preadmission hospitalization was 8.0 days (IQR=5-15). The average age of participants was 69.8 years (SD=9.3); 96.4% were male; 64.2% were white, 32.6% Black or African American, and 3.2% another race. The study population had significant disability, devices, wounds, and comorbidities (Table 1). Differences in participant race were evident across the three cohorts —Facility A was 81% White and 15% Black; Facility B was 59% White and 38% Black; Facility C was 33% White and 68% Black. Significant differences in baseline characteristics across the three cohorts were also detected for recent antibiotic use, wounds, PICC lines, length of preadmission hospitalization, length of stay in the study, median Katz score, and functional dependence in all ADLs assessed (Table 1). Compared with Facilities A and B, Facility C had participants with fewer PICC lines, shorter preadmission hospitalization, and shorter length of stay in the study, but higher ADL scores corresponding to greater functional dependence.
Recruitment	We conducted a multisite, prospective cohort study at three VA NHs from April 2021 to September 2023. Eligibility criteria included new admission to the NH and enrollment within three days, with written informed consent and Health Insurance Privacy and Portability Act (HIPAA) authorization obtained from the resident and/or their legally authorized representative. Exclusion criteria were non–English speaking and receipt of hospice care.
Ethics oversight	This study complies with all relevant ethical regulations, including the collection of written informed consent and Health Insurance Privacy and Portability Act (HIPAA) authorization from all participants or their legally authorized representative. The study was reviewed and approved by the VA Central Institutional Review Board (cIRB) and all three participating local site research oversight boards.

Note that full information on the approval of the study protocol must also be provided in the manuscript.

Field-specific reporting

Please select the one below that is the best fit for your research. If you are not sure, read the appropriate sections before making your selection.

☒ Life sciences ☐ Behavioural & social sciences ☐ Ecological, evolutionary & environmental sciences

For a reference copy of the document with all sections, see nature.com/documents/nr-reporting-summary-flat.pdf

Life sciences study design

All studies must disclose on these points even when the disclosure is negative.

Sample size	We based our sample size recruitment estimate on prior data from our research team, taking into account facility sizes and number of new admissions/month, and assuming a recruitment rate of approximately 50%. We determined our sample population from three VA NHs with a

total of 245 beds to be 200 in the first two years, with >600 follow-up visits.

Participants from three participating nursing homes were screened for eligibility via chart review by study staff and were approached in his or her room within the first three days of arriving at the NH. Eligibility criteria for study participation included new admission to the NH and enrollment within three days, with written informed consent and Health Insurance Privacy and Portability Act (HIPAA) authorization obtained from the resident and/or their legally authorized representative. Exclusion criteria were non–English speaking and receipt of hospice care. Because we had very few exclusion criteria, our study population is representative of the populations present at the three participating sites. We found no baseline or demographic differences between residents enrolled and those not enrolled. We enrolled 200 (37.7%) of 522 potentially eligible participants recently admitted to one of the three participating NHs. The main reasons for non-enrollment were resident refusal (n=269), and inability to obtain consent within three days of admission (n=61). Three enrolled participants were removed from the analysis due to absence of HIPAA authorization, for a total sample size of 197 participants—98 from Facility A, 57 from Facility B, and 42 from Facility C.

The primary study outcomes are described in the Clinical Data section. For outcome one, we used the entire sample of study participants (n=197, Tables 1 & 2) as well as a subset of participants with >1 in-room visit completed (n=182, Figures 1 & 2) to analyze MDROs present on baseline and at discharge (i.e., the last in-room study visit). To estimate new MDRO acquisition during the NH stay, we limited the analysis to participants with at least one follow-up in-room or interactive visit after baseline (n=185, Table 4). For outcome two, we assessed transmission events during observed interactive visits only (up to five per participant) and used n=135 participants who had at least one interactive visit completed (Table 5 & Figure 3). For outcome three, we used WGS to establish whether isolates involved in transmission events shared a clonal origin. We used n=14 participants who had at least one transmission event occur during an interactive visit and had sequencing completed (Figure 4).

Data exclusions

Three enrolled participants were removed from the analysis due to absence of HIPAA authorization, for a total sample size of 197 participants—98 from Facility A, 57 from Facility B, and 42 from Facility C.

Participant characteristics were described both for the overall cohort and stratified by facility (Table 1). We examined risk factors for baseline MDRO colonization and subsequent acquisition of new MDROs using logistic regression models. Covariates for the model were selected based on a univariate screening process (Supplementary Tables 1 and 2), with a more liberal selection threshold of $p = 0.1$. This threshold is more inclusive than the $p = 0.05$ commonly used for hypothesis testing, allowing for the identification of potentially important covariates that might otherwise be overlooked. Both univariate (Supplementary Tables 1 and 2) and multivariable (Table 3) analyses were conducted using participants with complete data for all risk factors.
Of the 197 residents initially included in the study, data on baseline MDRO colonization were available for all, while data on race and hospital length of stay (LOS) were missing for 7 (3.6%) and 2 (1.0%) residents, respectively. Complete data were available for 188 (95.4%) residents (Supplementary Table 1). For the analysis of new MDRO acquisition, 181 residents who were not colonized with all three MDROs upon enrollment were included in the analysis. Among these, data on race were missing for 7 (3.9%) and on hospital LOS for one resident, yielding complete data for 173 (95.6%) residents (Supplementary Table 2).

Replication

The main findings of this study were reproduced several times by the research team with successful results.

Randomization

There was no experimental randomization of participants into groups.

Blinding

No group allocation was performed due to the observational nature of this study. Investigators performing all microbiological testing and sequencing testing did not have knowledge of the clinical characteristics of participants corresponding to the samples at the time of testing.

Reporting for specific materials, systems and methods

We require information from authors about some types of materials, experimental systems and methods used in many studies. Here, indicate whether each material, system or method listed is relevant to your study. If you are not sure if a list item applies to your research, read the appropriate section before selecting a response.

Materials & experimental systems

n/a	Involved in the study	
☒	☐	Antibodies
☒	☐	Eukaryotic cell lines
☒	☐	Palaeontology and archaeology
☒	☐	Animals and other organisms
☐	☒	Clinical data
☒	☐	Dual use research of concern
☒	☐	Plants

Methods

n/a	Involved in the study	
☒	☐	ChIP-seq
☒	☐	Flow cytometry
☒	☐	MRI-based neuroimaging

Clinical data

Policy information about clinical studies
All manuscripts should comply with the ICMJE guidelines for publication of clinical research and a completed CONSORT checklist must be included with all submissions.

Clinical trial registration

N/A – our study was observational and not randomized

Study protocol	We have reported our methods previously and provide citations to our prior work.
Data collection	We conducted a multisite, prospective cohort study at three VA NHs from April 2021 to September 2023. Eligibility criteria included new admission to the NH and enrollment within three days, with written informed consent and Health Insurance Privacy and Portability Act (HIPAA) authorization obtained from the resident and/or their legally authorized representative. Exclusion criteria were non–English speaking and receipt of hospice care. We followed enrolled participants for up to three months or until discharge, whichever came first. Two different types of visits were completed with each participant: 1) regularly-scheduled in-room visits, which were conducted on the day of study enrollment, weekly for one month, and monthly thereafter for a maximum of three months (i.e., days 0, 7, 14, 21, 30, 60, 90); and 2) up to five unscheduled interactive visits, including but not limited to therapy gym visits for physical or occupational therapy, dialysis unit visits, radiology visits for x-rays, ophthalmology visits for routine eye appointments, radiation/oncology visits, and dining room visits for meals or recreation/activities. The frequency of interactive visits was not predetermined (as was the case with in-room visits). Participant hands, nares, groin, and seven environmental surfaces were swabbed during 758 regularly scheduled in-room visits; participant hands, healthcare personnel hands, and equipment were swabbed during 345 unscheduled interactive visits.
Outcomes	Outcomes of interest were as follows: 1) prevalence of longitudinal changes in MDRO colonization, including new acquisitions and spontaneous loss of MDROs; 2) MDRO transmission events during various interactive visits, including identification of interaction types associated with higher transmission rates; and 3) utilization of WGS to confirm sources of MDRO transmission during interactive visits.<br>Measurements:<br>Outcome 1: prevalence of MDROs (including MRSA, VRE, or R-GNB) on participant and in participant room at study baseline, at study discharge, and at any time during the study; prevalence of new acquisitions of MDROs (i.e., MRSA, VRE, or R-GNB) on participant and in participant room during the study; prevalence of spontaneous loss of MDROs (i.e., MRSA, VRE, or R-GNB) on participant and in participant room during the study; and risk factors for baseline MDRO colonization and new MDRO acquisition.<br>Outcome 2: prevalence of transmission events during different types of interactive visits (number of transmissions / number of interactive visits attended).<br>Outcome 3: within a subset of the population who had at least one transmission event occur during an interactive visit and had sequencing completed, prevalence of identical source and destination surfaces strains; prevalence of identical destination strains and those found at earlier, in-room visits (when a source within the interactive visit cannot be determined); prevalence of identical strains among colonized or contaminated patients or surfaces that are only swabbed one time, and thus transmission is not able to be assessed by our definition.<br>Definitions:<br>MDRO colonization - as any patient body site (nares, groin, or hand) colonized with any MDRO (MRSA, VRE, or R-GNB).<br>MDRO contamination - any environmental surface (bed control<br>New acquisition - at in-room visits only, when a participant or a participant's environment starts off negative for any MDRO at baseline, but becomes positive for any MDRO at some point during follow-up in-room visits.<br>Transmission - at interactive visits only, when a participant hand or surface is MDRO-negative before any use or contact, but becomes positive for any MDRO following contact with a hand or surface.<br>Source - Where a transmission originates from; i.e., a participant hand or surface that is colonized or contaminated first within the same interactive visit, and appears to pass the organism to a destination surface.<br>Destination Surface - Where a transmission ends up; i.e., a participant hand or surface that is not colonized or contaminated before use/contact, but becomes colonized or contaminated with any MDRO after use/contact.

Plants

Seed stocks	N/A
Novel plant genotypes	N/A
Authentication	N/A
